# Underweight Full-Term Indian Neonates Show Differences in Umbilical Cord Blood Leukocyte Phenotype: A Cross-Sectional Study

**DOI:** 10.1371/journal.pone.0123589

**Published:** 2015-04-21

**Authors:** Deepak K. Rathore, Deepa Nair, Saimah Raza, Savita Saini, Reeta Singh, Amit Kumar, Reva Tripathi, Siddarth Ramji, Aruna Batra, Kailash C. Aggarwal, Harish K. Chellani, Sugandha Arya, Neerja Bhatla, Vinod K. Paul, Ramesh Aggarwal, Nidhi Agarwal, Umesh Mehta, Shailaja Sopory, Uma Chandra Mouli Natchu, Shinjini Bhatnagar, Vineeta Bal, Satyajit Rath, Nitya Wadhwa

**Affiliations:** 1 Pediatric Biology Centre, Translational Health Science and Technology Institute, Gurgaon, Haryana, India; 2 Department of Pediatrics, All India Institute of Medical Sciences, New Delhi, India; 3 Department of Obstetrics & Gynecology, Maulana Azad Medical College, New Delhi, India; 4 Department of Neonatology, Maulana Azad Medical College, New Delhi, India; 5 Department of Obstetrics & Gynecology, Vardhman Mahavir Medical College & Safdarjung Hospital, New Delhi, India; 6 Department of Pediatrics, Vardhman Mahavir Medical College & Safdarjung Hospital, New Delhi, India; 7 Department of Obstetrics & Gynecology, All India Institute of Medical Sciences, New Delhi, India; 8 Department of Obstetrics & Gynecology, General Hospital, Gurgaon, Haryana, India; 9 Department of Pediatrics, General Hospital, Gurgaon, Haryana, India; 10 National Institute of Immunology, Aruna Asaf Ali Marg, New Delhi, India; University of Birmingham, UNITED KINGDOM

## Abstract

**Background:**

While infections are a major cause of neonatal mortality in India even in full-term neonates, this is an especial problem in the large proportion (~20%) of neonates born underweight (or small-for-gestational-age; SGA). One potential contributory factor for this susceptibility is the possibility that immune system maturation may be affected along with intrauterine growth retardation.

**Methods:**

In order to examine the possibility that differences in immune status may underlie the susceptibility of SGA neonates to infections, we enumerated the frequencies and concentrations of 22 leukocyte subset populations as well as IgM and IgA levels in umbilical cord blood from full-term SGA neonates and compared them with values from normal-weight (or appropriate-for-gestational-age; AGA) full-term neonates. We eliminated most SGA-associated risk factors in the exclusion criteria so as to ensure that AGA-SGA differences, if any, would be more likely to be associated with the underweight status itself.

**Results:**

An analysis of 502 such samples, including 50 from SGA neonates, showed that SGA neonates have significantly fewer plasmacytoid dendritic cells (pDCs), a higher myeloid DC (mDC) to pDC ratio, more natural killer (NK) cells, and higher IgM levels in cord blood in comparison with AGA neonates. Other differences were also observed such as tendencies to lower CD4:CD8 ratios and greater prominence of inflammatory monocytes, mDCs and neutrophils, but while some of them had substantial differences, they did not quite reach the standard level of statistical significance.

**Conclusions:**

These differences in cellular lineages of the immune system possibly reflect stress responses in utero associated with growth restriction. Increased susceptibility to infections may thus be linked to complex immune system dysregulation rather than simply retarded immune system maturation.

## Introduction

Neonatal mortality is a major contributor of under-five mortality globally [1]. This is particularly prominent in low- and-middle income countries. India’s high neonatal mortality (32/1000 live births) contributes substantially to its infant mortality (47/1000 live births) [2]. Approximately one-third of neonates born in India have a low birth weight [3], and neonatal mortality in India is 30% higher in neonates with mild growth retardation and 183% higher in neonates with severe growth retardation [4].

One major cause of neonatal mortality in India is serious systemic infection [3]. The immune system in neonates has been shown to be quantitatively and qualitatively distinct and to respond differently from the adult immune system, possibly contributing to greater neonatal susceptibility to infections in comparison to adults [5–7]. However, the development and maturation of the human immune system in the neonatal period is still incompletely understood. While some studies have characterized the major hematopoietic cell lineages in the full-term umbilical cord blood such as monocytes, lymphocytes, granulocytes and natural killer (NK) cells, and compared the profiles with those in adult blood [8,9] or in blood from premature neonates [10], detailed analyses of the neonatal immune cellular phenotype and function, especially with regard to newly defined subpopulations such as in monocytes [11] and B cells [12] are still lacking.

Moreover, while some information is available about the immune cell phenotype in full-term appropriate-for-gestational-age (AGA) neonates, there is hardly any information at all about the status of the immune system in full-term small-for-gestational-age (SGA) neonates, who account for nearly two-thirds of the SGA neonates born in India. Yet, it is plausible to hypothesize that the higher susceptibility of SGA neonates to infections [13,14] may be related to delayed immune system maturation or to other, more complex dysfunctionalities of the immune system associated with the intrauterine environment causing growth restriction. Almost the only evidence available so far is a comparison of the relative frequencies of CD4 and CD8 T cells in umbilical cord blood between 25 AGA and 25 SGA full-term neonates, showing that the CD4:CD8 ratio was significantly different between them [15]. Zinc has been reported to be involved as a micronutrient in the regulation of the differentiation of innate immune cellular lineages [16].The deficiency of zinc has been linked to a variety of immune defects [17,18] and we have been studying the effect of zinc on neonatal morbidity and mortality [19].

On this background, we describe and compare here phenotypes of leukocyte subset frequencies from umbilical cord blood in full-term SGA and AGA neonates. Our data show substantial differences in a number of immune cellular lineages between the two groups even when the SGA neonates are only mildly underweight with no other associated maternal or neonatal risk factors, and the nature of these differences indicates that they are likely to be complex correlates of the underweight situation rather than simply reflecting growth retardation in the immune system.

## Materials and Methods

### Study design

The estimated sample size to detect differences between SGA and AGA neonates in this cross-sectional study was calculated based on data from an earlier study in an Indian setting [15] and our preliminary data. The SGA neonate sample size to detect CD4:CD8 ratio differences between AGA and SGA neonates was 50, and the sample size for robust descriptive characterization of the immune cellular phenotype in AGA neonates was 455.

This cross-sectional study was done over April 2011-April 2013 at three hospitals in Delhi, the All India Institute of Medical Sciences (AIIMS), the Maulana Azad Medical College (MAMC), and the Vardhman Mahavir Medical College & Safdarjung Hospital (VMCC & SJH), and at one district hospital in the National Capital Region, the General Hospital, Gurgaon (GHG), coordinated by the Translational Health Science and Technology Institute (THSTI).

### Ethics Statement

The study protocol was approved by the Institute Ethics Committee, All India Institute of Medical Sciences, Institutional Ethics Committee, General Hospital Gurgaon, the ethics committees of Maulana Azad Medical College and Associated Hospitals, V. M. Medical College and Safdarjung Hospital and the Institutional Ethics Committee (Human Research), Translational Health Science and Technology Institute.

Written informed consent (or a thumb print from those who were illiterate) was taken (in the presence of a witness) for participation from eligible and willing participants upon detailed description.

### Participant eligibility, informed consent, and gestational age assessment

Pregnant women coming to the antenatal clinics any time at or after the 34^th^ week of gestation were screened for eligibility. Documented and witnessed informed consent for participation was taken from eligible and willing participants upon detailed description. Women likely to deliver at term by the vaginal route and with no evidence of multiple gestation, fetal congenital anomaly, documented major illness such as HIV, tuberculosis, thyroiditis, diabetes, or hypertension, were eligible for the study. Exclusion criteria included pregnancy-induced illness such as gestational diabetes, pre-eclampsia, or eclampsia, history of immunomodulatory medications, blood transfusion, infection in the last trimester lasting for three or more days, or hospitalization due to an infection at any time during pregnancy. Pregnant women who had opted for cord blood ‘banking’ were also excluded. Gestational age was estimated based on the date of ‘last menstrual period’. If this was not available, a dating ultrasound examination at the first antenatal visit was used to confirm the gestational age.

In order to ensure that the neonates were from a relatively homogenous group of women with minimal health-related risk factors, additional eligibility screening was done intra-partum and immediately after birth. Those who came in full-term normal labor, and had not developed any of the antenatal exclusion criteria in the interim were considered eligible. Neonates born by full-term vaginal delivery were assessed for ‘at-birth’ eligibility, to exclude those with one-minute APGAR of <7/10, birth weight < 1.8 kg, presence of Rh incompatibility or requirement for neonatal intensive care. The full-term neonates were classified as small-for-gestational-age (SGA) or appropriate-for-gestational-age (AGA) based on the birth weight and calculated gestational age at the time of birth using Lubchenco charts [20,21]. If the birth weight was between the 10th and 90th percentile for that gestational age, the neonate was classified as being AGA and if it was below 10th percentile for that gestational age, the neonate was classified as SGA. Weights were recorded on an electronic weighing scale (SECA, Hamburg, Germany) to the nearest 5 g.

### Cord blood collection

In eligible instances, ~25 ml of umbilical cord blood was collected from the cord vein by venipuncture at the cut end of the cord attached to the placenta once the placenta was delivered. Blood to be used for multi-color flow cytometric enumeration of leukocyte subsets was dispensed into heparinized collection tubes. Blood was also used for estimation of total leukocyte counts at THSTI using standard clinical hematology laboratory procedures. Sera from the remaining blood were aliquoted into 1 ml trace element-free vials for zinc, IgM, and IgA estimation and stored at -80°C till analysis.

### Serum IgM and IgA enzyme-linked immunosorbent assays (ELISAs)

Serum IgM and IgA levels were determined at THSTI using standard sandwich ELISA assays for IgM and IgA1/IgA2 (BD Biosciences, San Jose, CA, and Southern Biotech, Birmingham, AL). Purified human IgM and IgA (Sigma, St. Louis, MO) were used as standards.

### Serum zinc measurement

Serum zinc was measured with a flame furnace atomic absorption spectrophotometer (GBC Avanta, Dandenong, Australia) at AIIMS using standard procedures and with SERONORM (Sero AS, Billingstad, Norway) as the reference [22].

### Total leukocyte counts

Total leukocyte counts (TLCs) were done by standard clinical hematology laboratory procedures using WBC diluting fluid for counting in a modified Neubauer chamber (Sigma, St. Louis, MO 63103).

### Flow cytometric analysis of leukocytes

Erythrocytes from whole blood were lysed using ammonium chloride as described previously [23] and the leukocyte-enriched cell pellet was used for phenotypic analysis after washing with phosphate buffer saline (PBS). For each blood sample, five different staining cocktails of predetermined optimum concentrations of fluorochrome-labeled monoclonal antibodies (mAbs) (Table A in S1 File) were used for 3 million cells/well, cells were incubated on ice for 30 min in the dark, washed with PBS, fixed with 2% paraformaldehyde and data acquired on a flow cytometer (FACSCanto II or FACSAria III, BD Biosciences, San Jose, CA). FlowJo software (Tree Star, Ashland, OR) was used for analysis.

### Definition of cord blood leukocyte sub-populations

**Fig 1:**
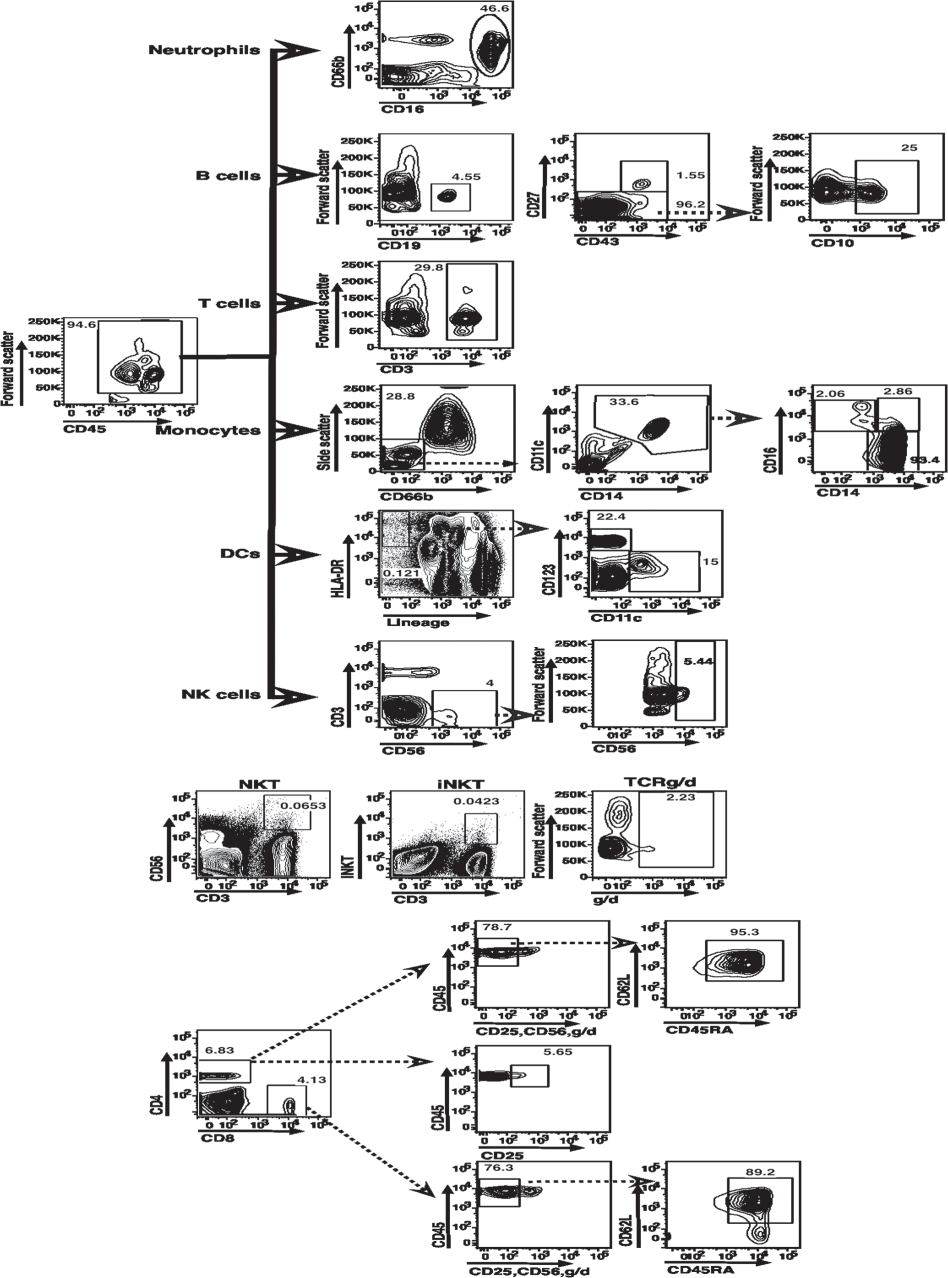
Flow cytometric-gating strategies for major cell lineages from cord blood. After primary gating for CD45, different cell subsets were gated as shown and percentage values for each subset obtained for further calculation of absolute numbers. I row—Neutrophil (CD45+CD66b+CD16+) gating. II row—CD45+CD19+ B cells were further gated for B1 B cells (CD45+CD19+CD27+CD43+), and naïve B cells (CD45+CD19+CD27-). Naïve B cells were further gated as immature naïve B cells (CD45+CD19+CD27-CD10+) and mature naïve B cells (CD45+CD19+CD27-CD10-). III row—CD45+CD3+ cells identified as T cells. IV row—Gating for monocytes (CD45+CD66b-CD14+CD11c+)[11], and among them, for patrolling monocytes (CD14dimCD16+), inflammatory monocytes (CD14+CD16+), and classical monocytes (CD14+CD16-). V row—Gating for dendritic cells (DCs) (CD45+‘lineage’- [CD3-CD14-CD16-CD19-CD20-CD56-] HLA-DR+CD11c+/CD123+), and among them, for myeloid DCs (mDCs; CD45+lineage-HLADR+CD11c+CD123-) and plasmacytoid DCs (pDCs; CD45+lineage-HLA-DR+CD11c-CD123+). VI row—Gating for NK cells (CD45+CD3-CD56+), and immature CD56-bright NK cells. VII row—CD45+ cells were further gated to identify NKT cells (CD45+CD3+CD56+), invariant NKT (iNKT) cells (CD45+ CD3+V alpha 24-J alpha 18 TCR+), and gamma-delta T cells (CD45+CD3+TCRg/d+). Bottom cluster (VIII, IX and X rows)—CD45+CD3+ T cells were further gated for putative regulatory T cells (CD45+CD3+CD4+CD25+), and classical T cells (CD45+CD3+CD56-CD25-TCRg/d-) which were sub-divided into CD4 and CD8 cells. CD4 and CD8 T cells were further characterized using CD45RA and CD62L as naïve (CD62L+CD45RA+) cells.

In the flow cytometric analyses, gating strategies were defined for identification of various leukocyte subsets (Fig 1). All leukocytes were identified using CD45 as a marker of bone marrow-derived cell lineages [24]. Further sub-populations were estimated as proportions of all CD45+ cells. Scatter properties were not used for further gating since all subsets measured were clearly identifiable without further scatter gating. The major lineages were defined as: neutrophils (CD45+CD66b+CD16+) [25], monocytes (CD45+CD14+CD11c+) [11], dendritic cells (DCs) (CD45+‘lineage’-[CD3-CD14-CD16-CD19-CD20-CD56-]HLA-DR+CD11c+/CD123+) [26], natural killer (NK) cells (CD45+CD3-CD56+) [27], B lymphocytes (CD45+CD19+) [12], and T lymphocytes (CD45+CD3+) [8].

Among monocytes, further sub-populations were defined as recently identified [11] patrolling monocytes (CD14dimCD16+), inflammatory monocytes (CD14+CD16+) and classical monocytes (CD14+CD16-) (Fig 1). DC sub-populations were gated as previously defined [26]; myeloid DCs (mDCs; CD45+lineage-HLADR+CD11c+CD123-) and plasmacytoid DCs (pDCs; CD45+lineage-HLA-DR+CD11c-CD123+) (Fig 1).

The B cell lineage was further distinguished [12] into B-1 B cells (CD45+CD19+CD27+CD43+), and naïve B cells (CD45+CD19+CD27-) (Fig 1). As expected, no memory B cells (CD45+CD19+CD27+CD43-) were identified in any cord blood samples. Naïve B cells were further subdivided, using CD10 expression [28], into immature naïve B cells (CD45+CD19+CD27-CD10+) and mature naïve B cells (CD45+CD19+CD27-CD10-) (Fig 1). Similarly, while our protocols could detect circulating plasmablasts (CD19+CD20-CD27+CD38+) [29], there were no such cells detected in cord blood samples, while putative B-1 B cells were readily detected. This is a matter of interest given the ongoing controversy regarding the nature of these putative B-1 B cells [30].

T cell subsets were also further defined (Fig 1). NKT cells were identified as CD45+CD3+CD56+. The invariant NKT (iNKT) cells, which use the T cell receptor (TCR)-alpha chain V alpha 24-J alpha18 [31], were defined among CD45+CD3+ cells using a specific mAb recognizing that polypeptide chain. The gamma-delta T cells were defined using a specific mAb (CD45+CD3+TCRg/d+). Regulatory T cells (Tregs) were identified as CD45+CD3+CD4+CD25+ [32]. This was a putative identification, since cells were not stained for FoxP3, and CD25 expression as a result of activation of classical CD4 T cells could not be entirely ruled out. Classical T cells were further defined as CD45+CD3+CD56-CD25-TCRg/d-, and sub-divided into CD4 and CD8 cells. CD4 and CD8 T cells were further characterized using CD45RA and CD62L into naïve (CD62L+CD45RA+) cells. While our protocols could detect ‘central’ and ‘effector’ memory as well as recently activated effector memory T cell populations, cord blood samples did not show the presence of any unambiguously non-naïve ‘memory’ T cells.

### Quality assurance

Before study initiation, study procedures were standardized such that all important variables such as birth weight, gestational age, maternal age and height, paternal age and education status, and newborn anthropometry were documented accurately. Collection of cord blood and methods of transportation of collected cord blood samples was standardized across the research staff to minimize measurement errors. Study supervisor physicians were trained with print and video material on antenatal, intra-partum and newborn assessment using standard protocols to ensure uniformity across study sites. Role-plays were done to train the research staff for providing information and taking written informed consent from pregnant women. Regular standardization exercises for clinical variables and laboratory procedures were conducted among the research team members to minimize intra-observer and inter-observer variability within and across the four hospitals.

### Data management and statistical analysis

Site supervisors checked all completed case report forms twice before they were sent for interactive double data entry in Microsoft Access (Version 2007, Microsoft Corporation, USA) with in-built logic, range, and consistency checks. Analyses were done as for a cross-sectional study using STATA (Version 12.0, Stata Corp, College Station, Texas, USA). The cell concentrations (per μl of blood) of the leukocyte subset populations were estimated from their frequencies and the total leukocyte count values. For normally distributed cell concentration or frequency data, mean and standard deviation values are presented. Wherever the cell concentration or frequency data were skewed, they are presented as median (IQR) and as box plot reporting medians and 95% confidence intervals; appropriate transformations were done and values are presented as geometric means and 95% confidence intervals.

We looked for differences in the frequencies as well as cell concentrations of different cell lineages and their subpopulations in the cord blood between the two groups of AGA and SGA neonates using ANCOVA. We also examined whether adjusting for *a priori*-decided potential confounding factors such as maternal age and gender of neonate modified the results. Differences between the two groups were examined both by probability and by effect size.

## Results

### Characteristics of the participant population

A total of 1562 pregnant women visiting antenatal clinics were screened for inclusion criteria. At the time of delivery, 541 of them were eligible and had consented for collection of the cord blood (Figure A in S1 File). Due to technical failures, only 502 of 541 cord blood samples could be analyzed, comprising of 50 SGA and 452 AGA neonates. Participant characteristics were recorded and tabulated (Table 1). Mean maternal age was 24.3 years. There were no unexpected differences in the features of the two groups, except that 64% SGA neonates were female despite an overall proportion of 49.2%. The mean birth weight was only moderately less in the SGA group (SGA, 2.32 kg; AGA, 2.96 kg), indicating that SGA neonates in this study did not have severe growth restriction. The mean gestational age of the neonates was similar between the two groups.

**Table 1:** Characteristics of participant population.

Characteristics ^*a*^		Total	SGA group	AGA group
		n = 502	n = 50	n = 452
Parental				
Mother’s age, (years)		24.3 (3.4)	23.7 (3.7)	24.4 (3.4)
Father’s age, (years)		28.2 (3.7)	27.5 (3.6)	28.3 (3.8)
		n = 411	n = 38	n = 373
Mother’s intrapartum weight, kg		58.0 (9.0)	52.6 (7.2)	58.5 (9.0)
		n = 454	n = 31	n = 349
Mother’s height, cm		152.2 (4.8)	152.2 (5.0)	152.2 (4.8)
Mother’s education status, (in years)		9.0 (4.0)	9.0 (3.0)	9.0 (4.0)
Father’s education status (in years)		10.0 (4.0)	10.0 (4.0)	10.0 (4.0)
Neonatal				
Female	n (%)	247 (49.2)	32 (64.0)	215 (47.6)
Gestational age at birth, (weeks)		39 (1.1)	39 (0.98)	39 (1.1)
Birth weight, kg		2.9 (0.4)	2.32 (0.20)	2.96 (0.36)
		n = 497	n = 49	n = 448
Length, cm		49.97 (1.8)	48.9 (1.1)	50.1 (1.9)
		n = 497	n = 49	n = 448
Head circumference, cm		34.2 (1.2)	33.7 (1.2)	34.3(1.2)
		n = 494	n = 50	n = 444
Cord blood serum zinc level, μg/dL^***b***^		79.2 (18.3)	80.9 (16.2)	79.1 (18.5)

^*a*^ All values are Mean (SD) except where specified

^*b*^ To convert zinc in μg/dL to SI unit (μmol/L) multiply by 0.153

SGA, small for gestational age (birth weight below the 10th centile or 2SD below mean for GA of reference/normal birth curves); AGA, appropriate for gestational age (birth weight between the 10^th^ and 90^th^ centile for GA of reference/normal birth curves).

All leukocyte subsets were estimated both by frequencies and as absolute concentrations per μL of blood using the TLC values to represent the concentration of total CD45+ leukocytes, and the comparative data for all subsets were evaluated for both statistical significance with and without adjustment for maternal age and neonatal gender, as well as for the effect size of differences between AGA and SGA neonates for each parameter (Tables 2–3; Tables B-C in S1 File).

**Table 2:** Comparison of absolute concentrations of immune markers between term SGA and AGA newborns.

Immune markers ^*a*^	SGA group	AGA group	ERC	Adjusted ERC ^*b*^
			(95% CI)	(95% CI)
			*P*	*P*
Total leukocyte count	n = 50	n = 452		
Geometric Mean (95% CI)	17345 (16119, 18664)	16781 (16370, 17202)	1.034 (0.956, 1.118)	1.024 (0.947, 1.108)
Median (IQR)	17825 (15100, 21050)	16650 (13725, 20125)	0.407	0.550
Innate immune markers				
Dendritic cells (DCs)	n = 43	n = 419		
Geometric Mean (95% CI)	50 (40, 61)	56 (53, 59)	0.895 (0.743, 1.077)	0.898 (0.744, 1.084)
Median (IQR)	54 (28, 81)	56 (53, 82)	0.239	0.263
Plasmacytoid DCs (pDCs)	n = 23	n = 259		
Geometric Mean (95% CI)	6 (4, 9)	9 (8, 10)	0.704 (0.506, 0.980)	0.690 (0.494, 0.964)
Median (IQR)	9 (3, 13)	10 (6, 14)	0.038	0.030
mDC:pDC ratio	n = 23	n = 259		
Geometric Mean (95% CI)	1.46 (1.09, 2.00)	0.95 (0.88, 1.03)	1.541 (1.164, 2.040)	1.557 (1.173, 2.068)
Median (IQR)	1.42 (0.82, 2.18)	0.96 (0.61, 1.42)	0.003	0.002
Natural Killer (NK) cells	n = 50	n = 451		
Geometric Mean (95% CI)	941 (813, 1089)	768 (730, 807)	1.226 (1.046, 1.437)	1.235 (1.052, 1.449)
Median (IQR)	937 (644, 1288)	785 (522, 1124)	0.012	0.010
CD56bright NK cells	n = 50	n = 450		
Geometric Mean (95% CI)	73 (60, 89)	62 (59, 66)	1.176 (0.982, 1.409)	1.199 (1.000, 1.437)
Median (IQR)	61 (48, 142)	63 (42, 94)	0.078	0.050
Inflammatory monocytes	n = 48	n = 445		
Geometric Mean (95% CI)	64 (54, 76)	55 (51, 58)	1.173 (0.965, 1.426)	1.167 (0.960, 1.420)
Median (IQR)	68 (44, 97)	55 (36, 84)	0.108	0.121
Innate like adaptive immune markers				
iNKT cells	n = 17	n = 170		
Geometric Mean (95% CI)	4.8 (3.2, 7.3)	4.1 (3.6, 4.7)	1.174 (0.754, 1.829)	1.189 (0.759, 1.865)
Median (IQR)	6.0 (3.6, 7.8)	4.7 (2.5, 7.8)	0.476	0.448
TCR γδ cells	n = 49	n = 448		
Geometric Mean (95% CI)	92 (78, 108)	103 (97, 109)	0.887 (0.740, 1.064)	0.884 (0.736, 1.062)
Median (IQR)	97 (61, 126)	106 (69, 162)	0.196	0.188
B1B cells	n = 50	n = 449		
Geometric Mean (95% CI)	5 (3, 6)	5 (5, 5)	0.906 (0.701, 1.171)	0.885 (0.684, 1.146)
Median (IQR)	5 (2, 9)	5 (3, 9)	0.452	0.355
Adaptive immune markers				
CD4:CD8 T cell ratio	n = 50	n = 447		
Geometric Mean (95% CI)	1.85 (1.65, 2.07)	2.02 (1.96, 2.09)	0.914 (0.821, 1.017)	0.901 (0.809, 1.003)
Median (IQR)	1.91 (1.43, 2.38)	2.02 (1.60, 2.53)	0.100	0.056
Naïve CD4:CD8 T cell ratio	n = 50	n = 447		
Geometric Mean (95% CI)	1.95 (1.75, 2.18)	2.13 (2.05, 2.21)	0.914 (0.813, 1.028)	0.903 (0.803, 1.016)
Median (IQR)	1.97 (1.48, 2.46)	2.14 (1.65, 2.78)	0.135	0.090
Naïve B cells	n = 50	n = 449		
Geometric Mean (95% CI)	429 (352, 522)	473 (444, 503)	0.907 (0.746, 1.104)	0.894 (0.734, 1.088)
Median (IQR)	502 (252, 643)	475 (314, 730)	0.331	0.262
CD10+ Naïve B cells	n = 50	n = 440		
Geometric Mean (95% CI)	96 (76, 120)	116 (108, 124)	0.827 (0.664, 1.030)	0.815 (0.654, 1.017)
Median (IQR)	122 (63, 157)	123 (73, 186)	0.090	0.070
Immunoglobulin				
IgM, mg/dL^*c*^	n = 54	n = 468		
Geometric Mean (95% CI)	8.39 (7.11, 9.91)	7.05 (6.69, 7.44)	1.189 (1.007, 1.405)	1.185 (1.002, 1.400)
Median (IQR)	7.39 (5.89, 11.37)	7.15 (4.52, 10.56)	0.041	0.048

^*a*^ All immune marker (IM) variables have been log transformed

^***b***^ Adjusted for pre-specified factors: maternal age & newborn gender

^*c*^ To convert IgM in mg/dL to SI unit (mg/L) multiply by 10

SGA, small for gestational age (birth weight below the 10^th^ centile or 2SD below mean for GA of reference/normal birth curves); AGA, appropriate for gestational age (birth weight between the 10^th^ and 90^th^ centile for GA of reference/normal birth curves); ERC, exponentiated regression coefficient, ordinary least square (OLS) regression analysis after log transformation of dependent variable (immune marker); 95% CI, 95 percent confidence interval.

**Table 3:** Comparison of relative frequencies of immune markers between term SGA and AGA newborns.

Immune markers ^*a*^	SGA group	AGA group	ERC	Adjusted ERC ^*b*^
			(95% CI)	(95% CI)
			*P*	*P*
Innate immune markers				
Neutrophils	n = 50	n = 450		
Geometric Mean (95% CI)	60.6 (57.9, 63.5)	57.5 (56.6, 58.4)	1.055 (1.006, 1.107)	1.053 (1.004, 1.106)
Median (IQR)	61.5 (53.7, 68.0)	58.4 (52.6, 64.7)	0.029	0.034
Dendritic cells (DCs)	n = 43	n = 419		
Geometric Mean (95% CI)	0.29 (0.24, 0.34)	0.33 (0.32, 0.35)	0.867 (0.742, 1.012)	0.880 (0.752, 1.029)
Median (IQR)	0.28 (0.17, 0.45)	0.34 (0.24, 0.46)	0.071	0.108
Myeloid DCs (mDCs)	n = 23	n = 259		
Geometric Mean (95% CI)	18.5 (14.6, 23.5)	14.2 (13.2, 15.2)	1.304 (1.021, 1.666)	1.276 (0.998, 1.632)
Median (IQR)	19.0 (13.1, 29.2)	14.5 (9.7, 22.0)	0.034	0.052
Plasmacytoid DCs (pDCs)	n = 23	n = 259		
Geometric Mean (95% CI)	12.6 (9.8, 16.2)	14.9 (13.8, 16.2)	0.846 (0.641, 1.117)	0.820 (0.620, 1.085)
Median (IQR)	14.0 (9.3, 17.2)	15.8 (10.2, 24.4)	0.238	0.163
Natural Killer (NK cells)	n = 50	n = 451		
Geometric Mean (95% CI)	5.42 (4.74, 6.19)	4.57 (4.36, 4.79)	1.185 (1.024, 1.372)	1.204 (1.040, 1.394)
Median (IQR)	5.58 (4.0, 7.1)	4.66 (3.36, 6.47)	0.023	0.013
Classical monocytes	n = 48	n = 445		
Geometric Mean (95% CI)	85.9 (84.1, 87.7)	87.6 (87.0, 88.1)	0.981 (0.962, 1.000)	0.979 (0.960, 0.999)
Median (IQR)	87.5 (81.8, 90.8)	88.7 (85.1, 91.5)	0.055	0.038
Inflammatory monocytes	n = 48	n = 445		
Geometric Mean (95% CI)	5.28 (4.44, 6.28)	4.49 (4.26, 4.74)	1.176 (0.990, 1.396)	1.177 (0.990, 1.400)
Median (IQR)	4.8 (3.63, 8.71)	4.6 (3.07, 6.66)	0.065	0.065
Adaptive immune markers				
CD4+ T cells	n = 50	n = 450		
Geometric Mean (95% CI)	9.1 (8.0, 10.3)	10.0 (9.6, 10.5)	0.905 (0.792, 1.033)	0.903 (0.790, 1.033)
Median (IQR)	8.87 (7.2, 12.6)	10.4 (7.7, 13.7)	0.138	0.136
B lymphocytes	n = 50	n = 449		
Geometric Mean (95% CI)	2.30 (1.89, 2.81)	2.74 (2.59, 2.91)	0.839 (0.699, 1.009)	0.837 (0.696, 1.007)
Median (IQR)	2.45 (1.52, 3.85)	2.83 (2.0, 4.06)	0.062	0.060

^*a*^ All immune marker variables have been log transformed

^***b***^ Adjusted for pre-specified factors: maternal age & newborn gender

SGA, small for gestational age (birth weight below the 10^th^ centile or 2SD below mean for GA of reference/normal birth curves); AGA, appropriate for gestational age (birth weight between the 10^th^ and 90^th^ centile for GA of reference/normal birth curves); ERC, exponentiated regression coefficient, ordinary least square (OLS) regression analysis after log transformation of dependent variable (immune marker); 95% CI, 95 percent confidence interval.

A comparison of the participant characteristics and the absolute concentrations as well as the relative frequencies of all immune markers between the four hospital sites (Tables D-F in S1 File) did not show any site-specific variation.

### Comparison of SGA and AGA cord blood leukocyte phenotypes: major cell lineages

**Fig 2:**
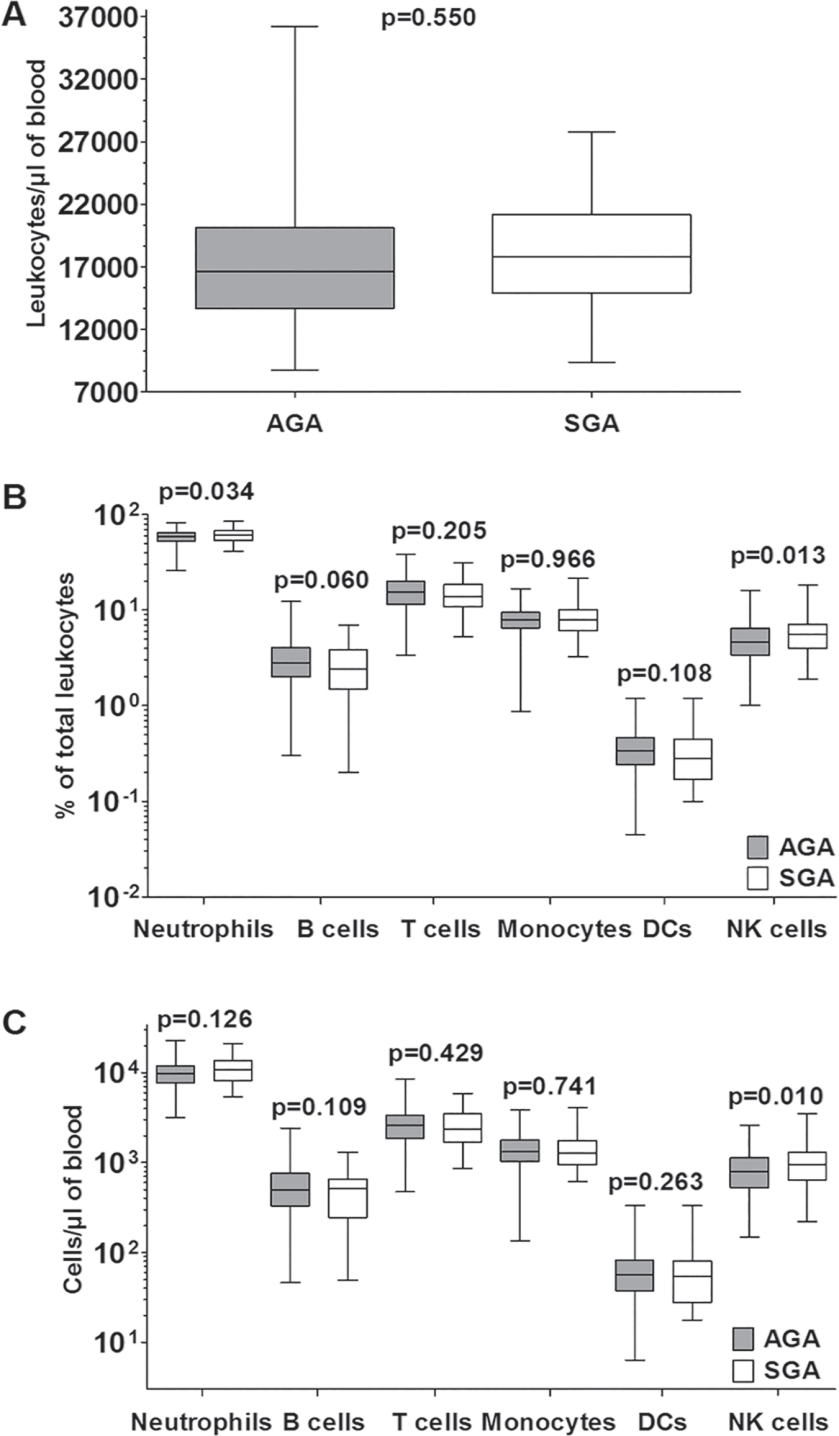
Neutrophils and NK cells are more prominent in SGA cord blood. (A) Absolute numbers of leukocytes in cord blood from AGA and SGA babies (median, interquartile range [IQR] and 95% confidence interval (CI). Adjusted p values (as mentioned in Tables 2–3; Tables B-C in S1 File) shown for each panel. (B) Major lineages in cord blood as % of total leukocytes (median, IQR and 95% CI) along with p values. (C) Absolute concentrations of major cell lineages in cord blood (median, IQR and 95% CI) along with p values.

The leukocyte cellularity of cord blood was not different between AGA and SGA neonates since their TLC values did not differ (Fig 2A). As a result, the frequency trends are similar to the cell concentration trends in all analyses below. SGA neonates tended to have higher frequencies of neutrophils (by ~5%, p = 0.034, Table 3), and lower frequencies of B cells (by ~16%, p = 0.060, Table 3) and DCs (by ~12%, p = 0.108, Table 3), although these differences were not as pronounced when cell concentrations were compared (Fig 2B and 2C). The frequencies and concentrations of T lineage cells and monocytes were not different between the two groups (Fig 2B and 2C). However, NK cell frequencies (by ~20%, p = 0.013) as well as concentrations (by ~24%, p = 0.010) were higher in SGA as compared to AGA neonates (Fig 2B and 2C).

### Comparison of SGA and AGA cord blood leukocyte phenotypes: B and T lymphocytic lineages

**Fig 3:**
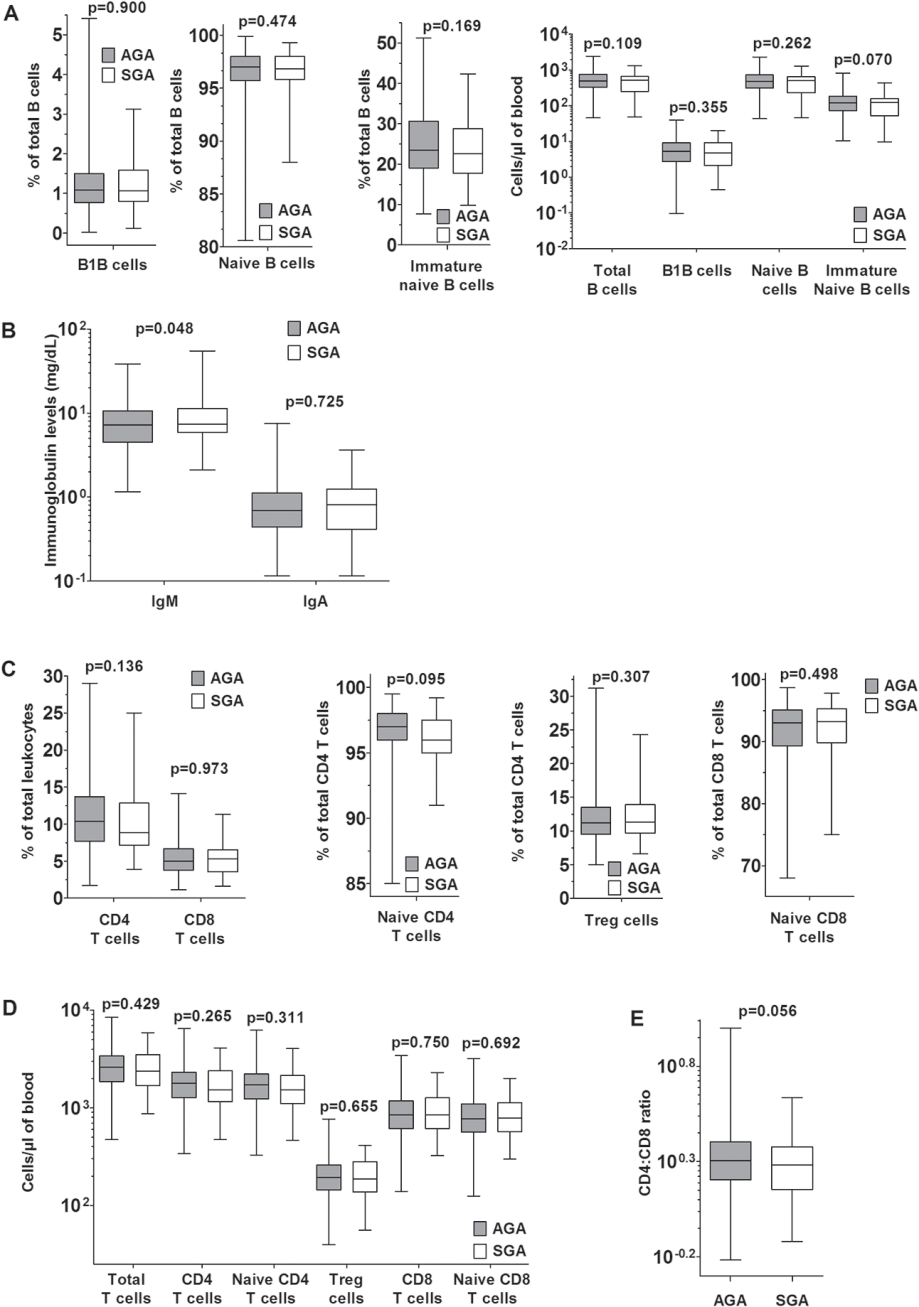
Immature naïve B cells, IgM levels and CD4:CD8 ratios show differences between AGA and SGA cord blood. (A) Comparison of proportions of B cell subsets and absolute concentrations (median, IQR and 95% CI) along with p values. (B) Comparison of absolute concentrations of IgM and IgA in cord blood (median, IQR and 95% CI) along with p values. (C) Comparison of proportions of T cell subsets (median, IQR and 95% CI) along with p values. (D) Comparison of absolute numbers of T cell subsets (median, IQR and 95% CI) along with p values. (E) Comparison of CD4:CD8 ratios (median, IQR and 95% CI) along with p value.

Within the classical B (CD19+) and T (CD3+) lineage cell populations, further sub-populations were also analyzed. In the B cell lineage, B-1 B cells (CD27+CD43+) showed no differences between the SGA and AGA groups (Fig 3A). There were no ‘memory’ B cells (CD9+CD27+CD43-) detected in any of the samples, nor were there any circulating plasmablasts (CD19+CD20-). The overwhelming majority of non-B-1 classical B cells were, as expected, naïve (CD27-CD43-). Within the naïve B cell population, CD10+ naïve B cells have been defined as immature transitional B cells recently emerged from the bone marrow [28,33,34] and are prominent in cord blood as expected and reported previously [28]. Interestingly, while the frequencies of these CD10+ naïve B cells were not different between SGA and AGA groups, their concentrations tended to be lower in SGA neonates but did not reach statistical significance (by ~19%, p = 0.070, Table 2), indicating that the B cell compartment may be somewhat more mature, if at all, in SGA than in AGA neonates.

As a measure of B cell functionality in vivo, we also estimated serum IgM and IgA levels in cord blood. Since neither maternal IgM nor IgA is known to cross placenta, both immunoglobulins in cord blood can be presumed to be of fetal origin [35–37]. There was no difference in IgA levels between SGA and AGA neonates (Fig 3B), but significantly higher levels of IgM were observed in SGA neonates (by ~19%, p = 0.048) (Fig 3B).

In the T lineage populations, AGA and SGA neonates did not show any differences in frequencies and concentrations of total CD4 and CD8 T cells and of putative regulatory T cells (CD4+CD25+), or in naïve CD4 or CD8 T cell concentrations (Fig 3C and 3D; Tables B-C in S1 File).

An earlier report from India [15], based on a very small sample size, had reported that the CD4:CD8 ratio in cord blood is different between AGA and SGA neonates, and we had calculated the sample size for our comparison based on those data. The CD4:CD8 ratio in our sample was lower in underweight neonates (by ~10%) with a p value of 0.056 (Fig 3E; Table 2), supporting, albeit weakly, evidence from reported literature.

### Comparison of SGA and AGA cord blood leukocyte phenotypes: cell types of the monocytic and DC lineage

**Fig 4:**
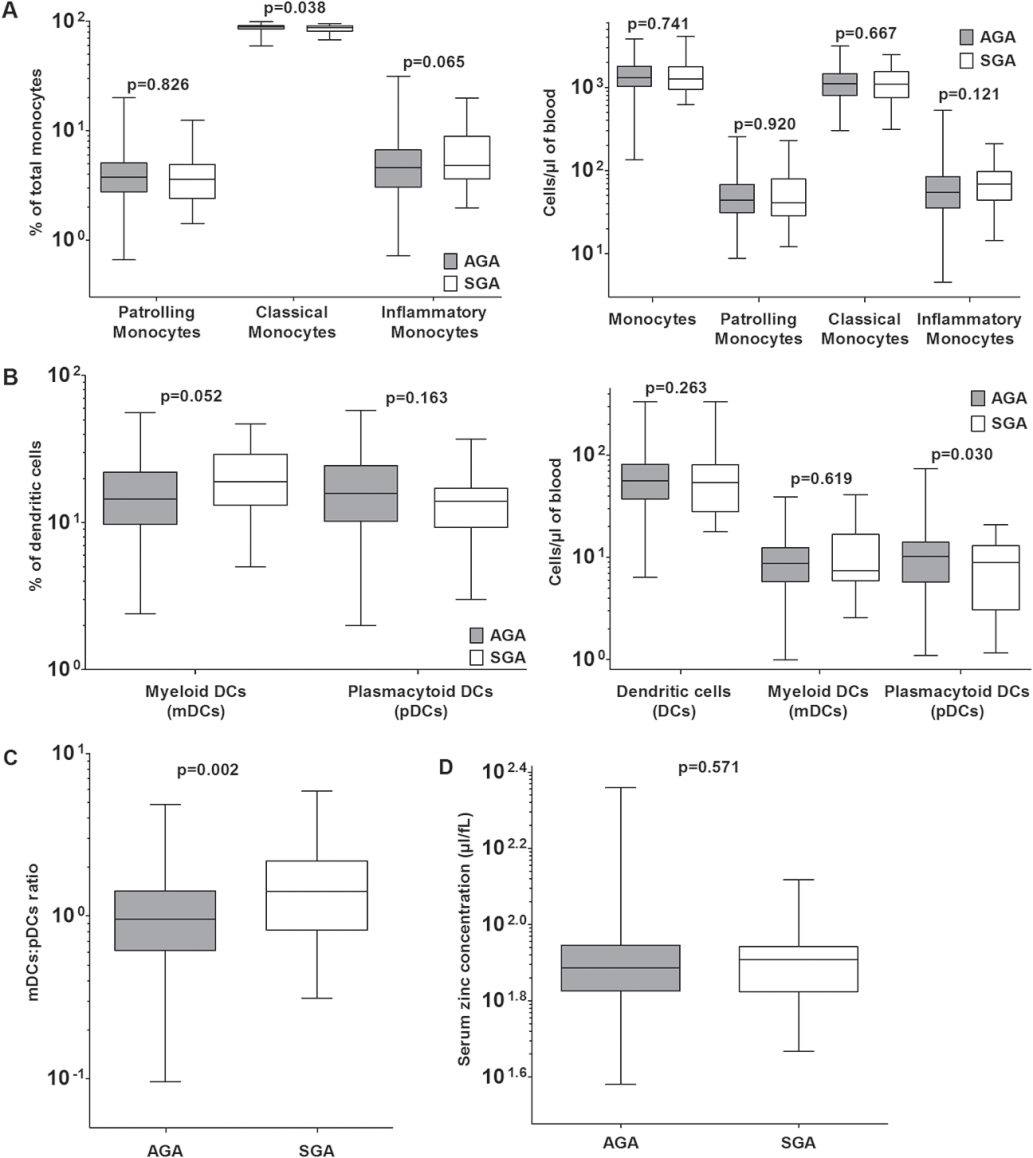
Classical monocytes, myeloid and plasmacytoid DCs as well as mDC: pDC ratios differ between AGA and SGA cord blood. (A) Comparison of proportions of monocyte subsets and absolute concentrations (median, IQR and 95% CI) along with p values. (B) Comparison of proportions of DC subsets and absolute concentrations (median, IQR and 95% CI) along with p values. (C) Comparison of mDC: pDC ratios in cord blood (median, IQR and 95% CI) along with p value. (D) Comparison of concentrations of zinc in cord blood (median, IQR and 95% CI) along with p value.

Functionally distinct monocyte subpopulations have been recently further subdivided in the human system based on cell-surface markers [11]. When these subsets were compared between SGA and AGA neonates, patrolling monocyte frequencies and concentrations were no different between AGA and SGA neonates whereas classical monocyte frequency was somewhat lower in SGA (Fig 4A). On the other hand, SGA neonates tended to have higher inflammatory monocyte frequencies (by ~18%, p = 0.065) and concentrations (by ~17%, p = 0.121) (Fig 4A). Among DC lineage cells, myeloid DC frequencies were higher (by ~28%, p = 0.052), and plasmacytoid DC numbers were significantly lower (by ~31%, p = 0.030) in SGA neonates (Fig 4B). As a result, the mDC: pDC ratio was substantially higher in SGA neonates (by ~56%, p = 0.002) (Fig 4C).

We observed no differences in the estimated serum zinc levels in cord blood from AGA and SGA neonates (Fig 4D).

### Comparison of SGA and AGA cord blood leukocyte phenotypes: innate-like non-classical T cell lineages

**Fig 5:**
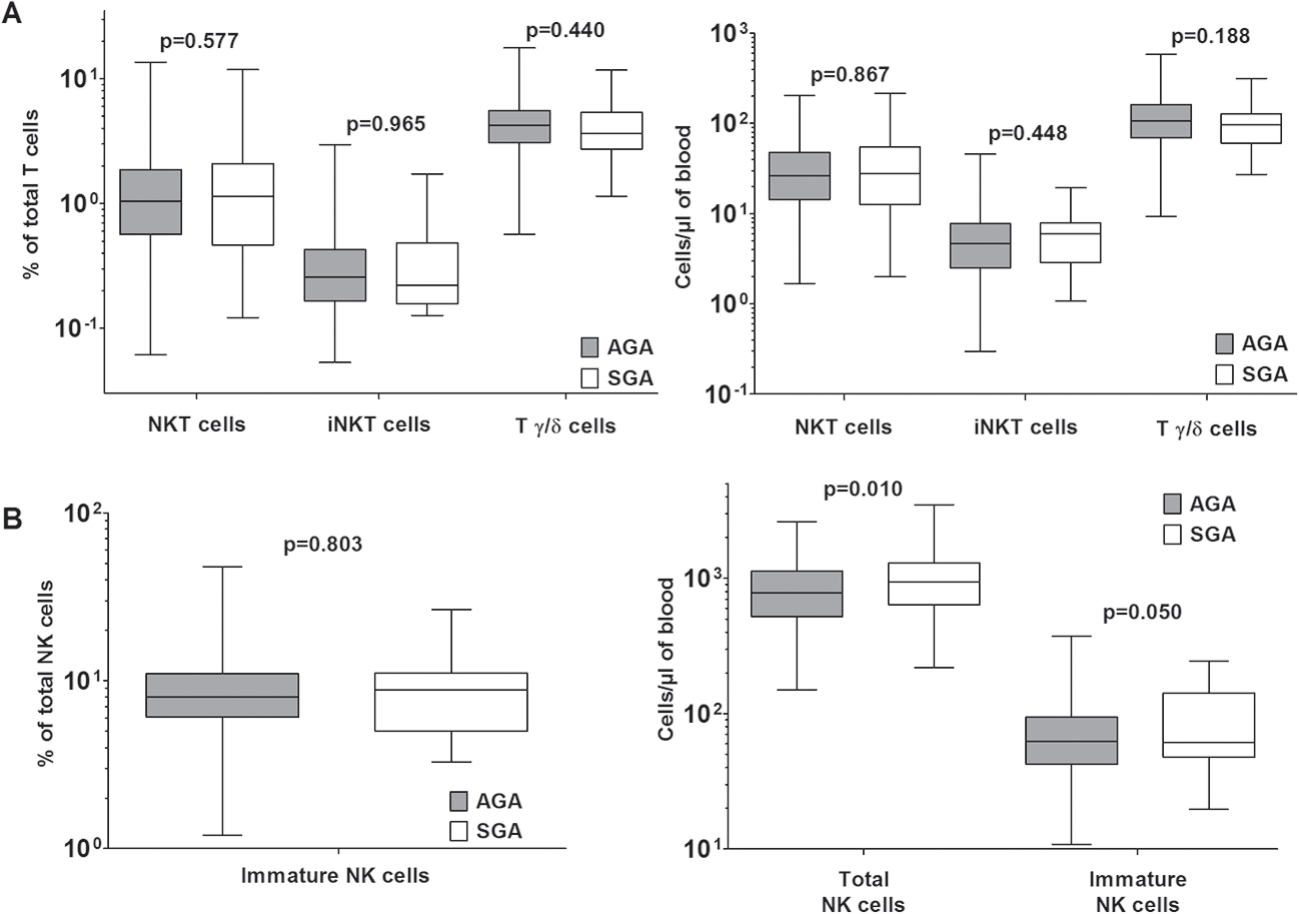
NK cells, but not innate-like T cells, show significant difference between AGA and SGA cord blood. (A) Comparison of proportions and absolute concentrations of NKT, iNKT and TCRγ/δ cells (median, IQR and 95% CI) along with p values. (B) Comparison of proportions and absolute numbers of immature NK cells (median, IQR and 95% CI) along with p values.

Among the immune cell lineages, we examined the frequencies and concentrations of the innate-like non-classical T cell lineages that have somatically diversified receptor repertoires of limited diversity and can mount effector responses upon primary activation. The frequencies and concentrations of NKT cells, invariant NKT (iNKT) cells and gamma/delta T cell receptor-expressing T cells were similar between AGA and SGA neonates (Fig 5A). It may be noted that, as shown above, the innate-like B cell lineage population of B-1 B cells also showed no differences between SGA and AGA neonates, but NK cell numbers were significantly higher in SGA neonates (Fig 5B). CD56 levels on NK cells did not show any evidence of relative NK cell immaturity in SGA neonates, since the frequencies of CD56bright immature NK cells (~2%) did not differ between the two groups (Fig 5B).

## Discussion

In this observational study we have characterized a large number of leukocyte cell lineages and their subpopulations in umbilical cord blood from a large series of full-term neonates, and carried out a comparison of the cord blood leukocyte phenotype between full-term AGA and SGA neonates to examine if SGA neonates show evidence of immunological immaturity. We report a number of differences between AGA and SGA neonates which together open up possibilities of looking for and analyzing potential immune dysfunction in the SGA neonates that constitute a major proportion of full-term born in the Indian subcontinent [38].

While the male-to-female gender ratio of 1.03 in our study is more consistent with the international ratio [39,40], studies in other Asian countries have demonstrated a higher male-to-female gender ratio of 1.2 [40]. Another observation in our study was that 64% SGA neonates were female despite an overall proportion of 49.2%. A study in China has shown similar trends where although the majority of adverse pregnancy and neonatal outcomes like caesarean delivery, preeclampsia, preterm birth, low birth weight and neonatal deaths have been noted to occur predominantly in males, interestingly, intra uterine growth restriction or SGA has been demonstrated to be more prevalent in females [40].

While there are reports, for example, of higher levels of pro-inflammatory cytokines in the cord blood of SGA neonates [41], it is quite possible that levels of small aqueous-phase molecules such as cytokines may show major fluctuations in cord blood, in part due to a maternal component and/or due to labor-induced stress. We have therefore primarily chosen the leukocyte phenotype as the basis to begin to evaluate differences, if any, in the status of the immune system between AGA and SGA neonates. While many cord blood leukocyte subpopulations show no differences between AGA and SGA neonates, we observe notable differences in some subsets.

Infection-associated morbidity and mortality has been reported to be higher in SGA as compared to AGA neonates in many locations across the world including India [42,43]. One explanation for this may be that, in association with the musculoskeletal growth retardation in SGA neonates, there may also be a delay in the maturation of the immune system. There are also reports that immune responses to vaccines may be poor in SGA neonates [44–47]. However, such deficits in responses of SGA neonates to vaccines are not uniformly found in all situations. Thus, while immune responses to hepatitis B vaccine have been found to be compromised [46], responses to other viral vaccines such as mumps, measles, rubella and varicella are not different in SGA neonates [48]. Similarly, while anti-tetanus antibody responses are compromised in SGA neonates, anti-pertussis responses are not [49]. A simple model of immaturity of the immune system, thus, may not be adequate to explain these complex findings.

There are also long-term consequences of the SGA status in terms of immunity-related morbidity starting with the ‘the small baby syndrome’ explained by the ‘thrifty phenotype hypothesis’ [50,51]. Epidemiological data show that low birth weight is associated with development of type II diabetes and metabolic syndrome in adulthood underpinned by a chronic inflammatory state, suggesting that the immune system may be differently programmed in underweight neonates. On the other hand, hypo functionality of the immune system in the form of a persistent inability to mount efficient responses to some vaccines has also been observed [52].

There are findings indicating that the immune system of prematurely born or SGA babies may be immature [53,54], with defects in both innate and adaptive immune components [54], including in their ability to mount responses to vaccines.

The ratio of CD4 to CD8 T cells (CD4:CD8 ratio) in the cord blood has been used as an indicator of maturity of the immune system. However, there are conflicting reports on the patterns observed in SGA neonates. A study from India shows that the CD4:CD8 ratio is lower in SGA babies [15], while another study from China reports that the ratio is higher in cord blood of babies with growth restriction [55]. A recent study showing SGA neonates have lower thymic volume as compared to AGA neonates [17] also suggests that thymic development is compromised in SGA babies thereby possibly adversely affecting the developmental maturity of the T cell compartment. It is notable that, while we do see a lower CD4:CD8 ratio in SGA neonates, the correlation is not as strong as that reported from India earlier [15]. Whether this is a result of the use of very stringent inclusion criteria for our group of SGA neonates remains to be seen.

Leukocyte and neutrophil granulocyte concentration in cord blood has been found to be lower in preterm (<32 weeks) SGA as compared to AGA neonates [56], with poor cytokine production from cord blood mononuclear cells of SGA neonates. Our data show SGA neonates to have a more prominent presence of inflammatory monocytes though not reaching statistical significance and some tendency to higher neutrophil leukocytes. If these cells are functionally normal, their prominence may be related to stress in utero.

Our findings show significantly greater frequencies and concentrations of cord blood NK cells in SGA neonates. A similar observation has been reported from preterm neonates [57]. Our data thus belie any simple expectation of relative immaturity of NK cell development in SGA neonates. There is previous evidence indicating that function may be compromised in cord blood NK cells as compared to NK cells from adult blood, and even more so in NK cells in cord blood from SGA neonates [58]. While we have not done functional analyses, we have looked at maturity of NK cells based on CD56 staining intensity [59] and our data do not show any differences in the proportions of mature NK cells between AGA and SGA neonates (data not shown). Whether the differences that we observed are because even phenotypically mature CD56-dull NK cells in SGA neonates are functionally compromised or because there are ethnic-geographical differences in SGA neonates needs further investigation.

Our data also show that pDC frequencies and concentrations in cord blood are lower in SGA neonates, with concomitantly higher mDC frequencies. It is known that pDCs are a major source of type I interferons and play a major role in anti-viral immunity [60]. Previous reports indicate that, as compared to adult pDCs, neonatal pDCs respond poorly to respiratory syncytial virus, a common pathogen encountered by neonates [61] and pDCs from premature neonates are worse than pDCs from full-term neonates in responses to both RSV and to TLR9 agonists [62]. It is thus possible that lower numbers of pDCs in SGA neonates further translate into compromised antiviral function. Neonatal mice show low levels of E2-2, a major pDC regulator gene product [63], suggesting a potential approach to study the differences in pDC maturation in SGA neonates. Some subsets of pDCs express indoleamine 2,3 oxidase (IDO) and function as anti-inflammatory cells and attenuate effector T cell responses [64], A deficit in such pDCs could thus potentially enhance inflammatory morbidity, although whether neonatal pDCs secrete type I interferons or not, and whether they belong to such an IDO-expressing subset is not currently known.

Another unusual finding emerging from our data is the higher levels of IgM but not IgA in SGA cord blood. We specifically chose to investigate IgM and IgA in cord blood since IgA and IgM do not cross placenta and hence are likely to be of fetal origin, while IgG and IgE from maternal blood are known to cross placenta [65,66]. While this could be a chance finding, it is noteworthy that the numbers and the level of maturation of the B cell lineage as measured by presence of CD10+ B cells tended to be lower in SGA neonates although not reaching statistical significance. This scenario, coupled with higher IgM levels, may be indicative of some stress-mediated enhancement or acceleration of B cell maturation and of the production of ‘natural’ antibodies [12].

Thus, our data show a pattern of lower numbers of pDCs, higher frequencies as well as numbers of NK cells, higher frequencies of neutrophils, higher mDC: pDC ratios, higher IgM concentrations in the absence of overt memory B cells in SGA neonates; and tendencies to lower CD4:CD8 ratios. Such a scenario may suggest a prominence of innate and innate-like adaptive immune components and delayed development of some adaptive cell lineages. This is not consistent with a simple hypothesis of delayed maturity of the immune system accompanying intrauterine growth restriction. In fact, the prominence of the early-response arms of the immune system could be hypothesized to be a response to intra-uterine stress, which is commonly argued to be a major accompaniment of growth restriction.

This altered immune state in SGA neonates could have two possible consequences in their responses to post-birth infections. On the one hand, if the prominent innate immune system is already activated to an extent, it may not be able to execute any further activation programs, resulting in delayed pathogen clearance. On the other hand, if the innate immune system is still capable of activation, then its prominence might mean that responses are exaggerated, leading to inflammation-mediated morbidity. This altered immune state rather than delayed maturation of the immune system in SGA neonates could help explain the higher morbidity and mortality in them.

A common limitation of studies on growth-restricted neonates is that neonatal growth restriction is commonly associated with other disorders, both maternal and/or fetal, such as maternal anemia, hypertensive disease, urinary tract or genital infection, HIV, fetal congenital heart defects, etc [67–71]. In such situations, it becomes difficult to determine if phenotypic alterations in SGA neonates, such as the ones we describe here, are associated with the fetal growth restriction per se or are more related to the co-morbidities. In order to minimize such limitations, we have used stringent inclusion/exclusion criteria to generate a homogenous participant population with minimal maternal health-related and environmental risk factors. Thus, the growth restriction in the full-term SGA neonates we have studied is relatively free of associated risk factors and is very mild (~15%). The fact that we still find robust and substantial differences in the immune cellular phenotypes of these SGA neonates strongly suggests that these differences could be attributed to being small at birth rather than to any associated co-morbidities in the mother or the neonate.

In conclusion, we report a large series on phenotypic characterization of cord blood from full term AGA and SGA neonates. Despite a small difference in the average weights of the two groups, we observe alterations in the immune phenotypes possibly reflecting stress responses in utero associated with growth restriction. Increased susceptibility to infections and poorer immune responses to vaccination may thus be linked to immune system dysregulation rather than to an immature immune system. More data on functional competence of the affected cellular subsets might help in further mechanistic characterization for the deficits in immune competence.

## Supporting Information

S1 File Figure A, Study profile. Flowchart showing screening and multi-step selection procedure for collection of information and cord blood. Table A, Cell surface markers used for fluorochrome-labeled monoclonal antibody cocktails. Table B, Comparison of absolute concentrations of immune markers between term SGA and AGA newborns. Table C, Comparison of relative frequencies of immune markers between term SGA and AGA newborns. Table D, Characteristics of participant population in the four hospital sites. Table E, Comparison of absolute concentrations of immune markers in term AGA newborns between the hospital sites. Table F, Comparison of relative frequencies of immune markers in term AGA newborns between the hospital sites.
(DOCX)
