# Supplementary material for: Underweight Full-Term Indian Neonates Show Differences in Umbilical Cord Blood Leukocyte Phenotype: A Cross-Sectional Study
Source: PLoS One. 2015 Apr 21;10(4):e0123589. doi: 10.1371/journal.pone.0123589 (PMC4405369; doi:10.1371/journal.pone.0123589)
Supplement: S1 File — Flowchart showing screening and multi-step selection procedure for collection of information and cord blood. Table A, Cell surface markers used for fluorochrome-labeled monoclonal antibody cocktails. Table B, Comparison of absolute concentrations of immune markers between term SGA and AGA newborns. Table C, Comparison of relative frequencies of immune markers between term SGA and AGA newborns. Table D, Characteristics of participant population in the four hospital sites. Table E, Comparison of absolute concentrations of immune markers in term AGA newborns between the hospital sites. Table F, Comparison of relative frequencies of immune markers in term AGA newborns between the hospital sites. (DOCX) [file pone.0123589.s001.docx]

**Supporting information**

**Figure A**

Screened pregnant women > 34 weeks gestation who came for a routine visit to antenatal clinic (n=1562)

Pregnant women eligible for antenatal enrolment (n=1062)

Pregnant women eligible and consent given (n=873)

Cord blood collected (n=541)

Delivered during non-working hours (n=310)

Intrapartum Exclusion (n=22)

Refused Consent (n=189)

**Excluded** (n=500)

Grand multipara mother (n=10), Multiple gestation (n=15), Gestational diabetes (n=63), Hypothyroidism (n=62), Preeclampsia/ Eclampsia (n=63), Fetal congenital anomaly (n=7), Others (n=280)

**Figure A Study profile.** Flowchart showing screening and multi-step selection procedure for collection of information and cord blood.

**Table A. Cell surface markers used for fluorochrome-labeled monoclonal antibody cocktails**

| **Cocktail- A** | |
| --- | --- |
| **Antibody (Clone)** | **Flurochrome** |
| CD45 (2D1) | APC-H7 |
| CD16 (3G8) | PE-Cy7 |
| CD66b (G10F5) | PerCP-Cy5.5 |
| CD14 (M5E2) | FITC |
| CD11c (B-ly6) | V450 |
| CD163 (GHI/61) | APC |
| **Cocktail- B** | |
| CD45 (2D1) | APC-H7 |
| CD3 (OKT-3) | PerCP-Cy5.5 |
| CD56 (NACAM16.2) | PE-Cy7 |
| CD4 (RPA-T4) | V450 |
| CD25 (M-A251) | FITC |
| TCR γ/δ (B1) | PE |
| Vα24 Jα18 TCR (6B11) | APC |
| **Cocktail- C** | |
| CD45 (2D1) | APC-H7 |
| CD4 (RPA-T4) | V450 |
| CD8 (RPA-T8) | PerCP-Cy5.5 |
| CD45RA (HI100) | PE-Cy7 |
| CD62L (DREG-56) | PE |
| TCR γ/δ (B1) | FITC |
| CD56 (NACAM16.2) | FITC |
| CD25 (M-A251) | FITC |
| **Cocktail- D** | |
| CD45 (2D1) | APC-H7 |
| CD19 (HIB19) | APC |
| CD20 (2H7) | PerCP-Cy5.5 |
| CD10 (HI10a) | PE-Cy7 |
| CD43 (10G7) | FITC |
| CD27 (M-T271) | V450 |
| **Cocktail- E** | |
| CD45 (2D1) | APC-H7 |
| Lineage 1 (SK7, 3G8, SJ25C1, L27, MφP9, NCAM16.2) | FITC |
| HLA-DR (L243) | PE-Cy7 |
| CD11c (B-ly6) | V450 |
| CD123 (7G3) | APC |

The following fluorochrome-labeled mAbs used on cell-surface markers: CD45, CD16, CD14, CD11c, CD56, CD25, CD3, CD4, CD8, TCRg/d, CD45RA, CD62L, CD19, CD20, CD27, Lineage (LIN: CD3, CD14, CD16, CD19, CD20, CD56), HLA-DR, CD123 were procured from BD Biosciences San Jose, CA; and CD66b, CD163, TCR Vα24 Jα18, CD43, CD10 were procured from BioLegend Inc. San Diego, CA.

**Table B. Comparison of absolute concentrations of immune markers between term SGA and AGA newborns**

| Immune markers *^a^* | SGA group | AGA group | ERC | Adjusted ERC *^b^* |
| --- | --- | --- | --- | --- |
|  |  |  | (95% CI) | (95% CI) |
|  |  |  | *P* | *P* |
| Total leukocyte count | n=50 | n=452 |  |  |
| Geometric Mean (95% CI) | 17345 (16119, 18664) | 16781 (16370, 17202) | 1.034 (0.956, 1.118) | 1.024 (0.947, 1.108) |
| Median (IQR) | 17825 (15100, 21050) | 16650 (13725, 20125) | 0.407 | 0.550 |
| Innate immune markers |  |  |  |  |
| Neutrophils | n=50 | n=450 |  |  |
| Geometric Mean (95% CI) | 10517 (9611, 11509) | 9649 (9357, 9951) | 1.090 (0.989, 1.201) | 1.079 (0.979, 1.189) |
| Median (IQR) | 10770 (8320, 13574) | 9810 (7751, 11946) | 0.081 | 0.126 |
| Myeloid DCs (mDCs) | n=23 | n=259 |  |  |
| Geometric Mean (95% CI) | 9 (7, 13) | 9 (8, 9) | 1.085 (0.817, 1.440) | 1.074 (0.809, 1.428) |
| Median (IQR) | 7 (6, 17) | 9 (6, 12) | 0.572 | 0.619 |
| Monocytes | n=50 | n=447 |  |  |
| Geometric Mean (95% CI) | 1352 (1206, 1516) | 1313 (1261, 1366) | 1.030 (0.909, 1.167) | 1.021 (0.901, 1.158) |
| Median (IQR) | 1267 (954, 1756) | 1314 (1031, 1792) | 0.640 | 0.741 |
| Patrolling monocytes | n=48 | n=445 |  |  |
| Geometric Mean (95% CI) | 45 (37, 54) | 45 (43, 48) | 0.998 (0.828, 1.204) | 1.010 (0.836, 1.219) |
| Median (IQR) | 41 (29, 77) | 44 (31, 67) | 0.985 | 0.920 |
| Classical monocytes | n=48 | n=445 |  |  |
| Geometric Mean (95% CI) | 1042 (902, 1204) | 1065 (1022, 1110) | 0.979 (0.856, 1.119) | 0.971 (0.848, 1.111) |
| Median (IQR) | 1100 (752, 1523) | 1103 (803, 1449) | 0.753 | 0.667 |
| Innate like adaptive immune markers | |  |  |  |
| NKT cells | n=50 | n=451 |  |  |
| Geometric Mean (95% CI) | 26 (20, 33) | 25 (23, 27) | 1.018 (0.790, 1.312) | 1.022 (0.792, 1.319) |
| Median (IQR) | 28 (13, 54) | 26 (14, 48) | 0.887 | 0.867 |
| Adaptive immune markers |  |  |  |  |
| T lymphocytes | n=50 | n=451 |  |  |
| Geometric Mean (95% CI) | 2370 (2076, 2705) | 2488 (2384, 2596) | 0.953 (0.832, 1.090) | 0.947 (0.826, 1.085) |
| Median (IQR) | 2370 (1694, 3510) | 2598 (1863, 3394) | 0.480 | 0.429 |
| CD4+ T cells | n=50 | n=450 |  |  |
| Geometric Mean (95% CI) | 1575 (1379, 1799) | 1685 (1614, 1759) | 0.935 (0.816, 1.071) | 0.925 (0.807, 1.061) |
| Median (IQR) | 1535 (1159, 2390) | 1784 (1277, 2317) | 0.331 | 0.265 |
| Naive CD4+ T cells | n=50 | n=448 |  |  |
| Geometric Mean (95% CI) | 1528 (1343, 1739) | 1628 (1559, 1700) | 0.939 (0.819, 1.076) | 0.931 (0.812, 1.069) |
| Median (IQR) | 1525 (1107, 2096) | 1732 (1229, 2224) | 0.364 | 0.311 |
| T regulatory cells | n=50 | n=449 |  |  |
| Geometric Mean (95% CI) | 186 (163, 213) | 191 (183, 199) | 0.976 (0.850, 1.120) | 0.969 (0.843, 1.113) |
| Median (IQR) | 186 (139, 277) | 193 (145, 259) | 0.728 | 0.655 |
| CD8+ T cells | n=50 | n=447 |  |  |
| Geometric Mean (95% CI) | 852 (751, 966) | 835 (798, 873) | 1.020 (0.886, 1.174) | 1.023 (0.888, 1.179) |
| Median (IQR) | 842 (611, 1259) | 844 (610, 1176) | 0.783 | 0.750 |
| Naïve CD8+ T cells | n=50 | n=447 |  |  |
| Geometric Mean (95% CI) | 783.6 (691.6, 887.9) | 764.3 (730.3, 799.8) | 1.025 (0.890, 1.182) | 1.029 (0.892, 1.187) |
| Median (IQR) | 782.3 (572, 1107.6) | 769.2 (560.9, 1089.1) | 0.729 | 0.692 |
| Immunoglobulin |  |  |  |  |
| IgA, mg/dL *^c^* | n=53 | n=459 |  |  |
| Geometric Mean (95% CI) | 0.74 (0.59, 0.94) | 0.71 (0.66, 0.75) | 1.047 (0.856, 1.280) | 1.037 (0.847, 1.270) |
| Median (IQR) | 0.81 (0.42, 1.16) | 0.69 (0.44, 1.11) | 0.654 | 0.725 |

*^a^* All immune marker variables have been log transformed

*^b^* Adjusted for pre-specified factors: maternal age & newborn gender

*^c^* To convert IgA in mg/dL to SI unit (mg/L) multiply by 10

SGA, small for gestational age (birth weight below the 10^th^ centile or 2SD below mean for GA of reference/normal birth curves); AGA, appropriate for gestational age (birth weight between the 10^th^ and 90^th^ centile for GA of reference/normal birth curves); ERC, exponentiated regression coefficient, ordinary least square (OLS) regression analysis after log transformation of dependent variable (immune marker); 95% CI, 95 percent confidence interval.

**Table C. Comparison of relative frequencies of immune markers between term SGA and AGA newborns**

| Immune markers *^a^* | SGA Group | AGA Group | ERC | Adjusted ERC *^b^* |
| --- | --- | --- | --- | --- |
|  |  |  | (95% CI) | (95% CI) |
|  |  |  | *P* | *P* |
| Innate immune markers |  |  |  |  |
| CD56bright NK cells | n=50 | n=450 |  |  |
| Geometric Mean (95% CI) | 7.87 (6.73, 9.21) | 8.09 (7.73, 8.47) | 0.973 (0.840, 1.128) | 0.981(0.846, 1.138) |
| Median (IQR) | 8.83 (5.17, 11.0) | 8.00 (6.10, 11.0) | 0.718 | 0.803 |
| Monocytes | n=50 | n=447 |  |  |
| Geometric Mean (95% CI) | 7.80 (7.04, 8.64) | 7.82 (7.59, 8.05) | 0.997 (0.908, 1.096) | 0.998 (0.908, 1.097) |
| Median (IQR) | 7.93 (6.1, 10.0) | 8.0 (6.52, 9.54) | 0.956 | 0.966 |
| Patrolling monocytes | n=48 | n=445 |  |  |
| Geometric Mean (95% CI) | 3.71 (3.18, 4.33) | 3.71 (3.52, 3.90) | 1.000 (0.849, 1.179) | 1.019 (0.865, 1.200) |
| Median (IQR) | 3.61 (2.44, 4.81) | 3.76 (2.76, 5.08) | 0.996 | 0.826 |
| Innate like adaptive immune markers | |  |  |  |
| NKT cells | n=50 | n=451 |  |  |
| Geometric Mean (95% CI) | 1.08 (0.82, 1.44) | 1.01 (0.93, 1.10) | 1.069 (0.818, 1.398) | 1.080 (0.824, 1.414) |
| Median (IQR) | 1.15(0.47, 2.07) | 1.05 (0.56, 1.86) | 0.624 | 0.577 |
| iNKT cells | n=17 | n=170 |  |  |
| Geometric Mean (95% CI) | 0.29 (0.20, 0.41) | 0.28 (0.25, 0.32) | 1.008 (0.685, 1.481) | 1.009 (0.683, 1.489) |
| Median (IQR) | 0.22 (0.16, 0.47 | 0.26 (0.17, 0.43) | 0.969 | 0.965 |
| TCR γδ cells | n=49 | n=448 |  |  |
| Geometric Mean (95% CI) | 3.89 (3.37, 4.50) | 4.14 (3.96, 4.34) | 0.939 (0.812, 1.087) | 0.944 (0.815, 1.093) |
| Median (IQR) | 3.66 (2.75, 5.34) | 4.24 (3.08, 5.52) | 0.398 | 0.440 |
| B1B cells | n=50 | n=449 |  |  |
| Geometric Mean (95% CI)  Median (IQR) | 1.02 (0.85, 1.23)  1.06 (0.8, 1.57) | 1.02 (0.97, 1.08)  1.08 (0.77, 1.5) | 0.997 (0.832, 1.195)  0.973 | 0.988 (0.823, 1.186)  0.900 |
| Adaptive immune markers |  |  |  |  |
| T lymphocytes | n=50 | n=451 |  |  |
| Geometric Mean (95% CI) | 13.6 (12.2, 15.3) | 14.8 (14.2, 15.4) | 0.921 (0.814, 1.042) | 0.923 (0.815, 1.045) |
| Median (IQR) | 13.7 (11.0, 18.5) | 15.4 (11.5, 20.0) | 0.192 | 0.205 |
| Naive CD4+ T cells | n=50 | n=448 |  |  |
| Geometric Mean (95% CI) | 96.1 (95.6, 96.6) | 96.5 (96.4, 96.7) | 0.995 (0.991, 1.001) | 0.995 (0.990, 1.001) |
| Median (IQR) | 96.0 (95.0, 97.5) | 97.0 (96.0, 98.0) | 0.092 | 0.095 |
| T regulatory cells | n=50 | n=449 |  |  |
| Geometric Mean (95% CI) | 1.06 (0.94, 1.21) | 1.14 (1.09, 1.19) | 0.934 (0.818, 1.066) | 0.933 (0.816, 1.066) |
| Median (IQR) | 1.05 (0.79, 1.50) | 1.14 (0.89, 1.55) | 0.308 | 0.307 |
| CD8+ T cells | n=50 | n=447 |  |  |
| Geometric Mean (95% CI) | 4.91 (4.36, 5.53) | 4.98 (4.78, 5.19) | 0.986 (0.867, 1.121) | 0.998 (0.877, 1.135) |
| Median (IQR) | 5.32 (3.53, 6.50) | 5.0 (3.78, 6.70) | 0.826 | 0.973 |
| Naïve CD8+ T cells | n=50 | n=447 |  |  |
| Geometric Mean (95% CI) | 92 (90.7, 93.4) | 91.1 (90.8, 93.4) | 1.005 (0.989, 1.022) | 1.006 (0.989, 1.023) |
| Median (IQR) | 93.3 (90.1, 95.3) | 93 (89.3, 95.1) | 0.537 | 0.498 |
| Naïve B cells | n=50 | n=449 |  |  |
| Geometric Mean (95% CI) | 96.4 (95.7, 97.1) | 96.6 (96.4, 96.8) | 0.998 (0.991, 1.005) | 0.997(0.991, 1.004) |
| Median (IQR) | 96.9 (96.0, 98.0) | 97.0 (95.7, 98.0) | 0.571 | 0.474 |
| CD10+ Naïve B cells | n=50 | n=440 |  |  |
| Geometric Mean (95% CI) | 22.2 (20.0, 24.7) | 23.6 (22.8, 24.4) | 0.943 (0.849, 1.046) | 0.929 (0.837, 1.032) |
| Median (IQR) | 22.6 (18.1, 28.8) | 23.5 (19.1, 30.5) | 0.266 | 0.169 |

*^a^* All immune marker variables have been log transformed

*^b^* Adjusted for pre-specified factors: maternal age & newborn gender

SGA, small for gestational age (birth weight below the 10^th^ centile or 2SD below mean for GA of reference/normal birth curves); AGA, appropriate for gestational age (birth weight between the 10^th^ and 90^th^ centile for GA of reference/normal birth curves); ERC, exponentiated regression coefficient, ordinary least square (OLS) regression analysis after log transformation of dependent variable (immune marker); 95% CI, 95 percent confidence interval.

**Table D. Characteristics of participant population in the four hospital sites**

| Characteristics *^a^* |  | Total | MAMC | SJH | AIIMS | GHG |
| --- | --- | --- | --- | --- | --- | --- |
|  |  | n=502 | n=276 | n=178 | n=21 | n=27 |
| Parental |  |  |  |  |  |  |
| Mother’s age, (years) |  | 24.3 (3.4) | 24.9 (3.5) | 23.6 (3.2) | 24.4 (3.6) | 23.4 (2.8) |
| Father’s age, (years) |  | 28.2 (3.7) | 28.4 (3.8) | 27.9 (3.5) | 29.0 (3.2) | 27.7 (4.4) |
|  |  | n=411 | n=256 | n=133 | n=19 | n=3 |
| Mother’s intrapartum weight, kg |  | 58.0 (9.0) | 58.3 (8.8) | 55.9 (8.3) | 66.6 (8.3) | 59.7 (18.5) |
|  |  | n=454 | n=272 | n=147 | n=20 | n=15 |
| Mother’s height, cm |  | 152.2 (4.8) | 152.5 (3.6) | 151.4 (6.1) | 155.9 (5.4) | 151.2 (6.5) |
| Mother’s education status, (in years) |  | 9.0 (4.0) | 9.0 (4.0) | 9.0 (5.0) | 12.0 (4.0) | 9.0 (5.0) |
| Father’s education status (in years) |  | 10.0 (4.0) | 10.0 (4.0) | 11.0 (4.0) | 13.0 (4.0) | 11.0 (3.0) |
| Neonatal |  |  |  |  |  |  |
| Female | n (%) | 247 (49.2) | 150 (54.3) | 74 (41.8) | 7 (33.3) | 16 (59.3) |
| Gestational age at birth, (weeks) |  | 39 (1.1) | 39 (1.0) | 39 (1.1) | 38 (1.1) | 39 (1.1) |
| Birth weight, kg |  | 2.9 (0.4) | 2.85 (0.38) | 2.95 (0.41) | 2.84 (0.40) | 3.07 (0.40) |
|  |  | n=497 | n=276 | n=178 | n=16 | n=27 |
| Length, cm |  | 49.97 (1.8) | 50.0 (0.9) | 49.8 (1.9) | 47.5 (3.6) | 51.8 (3.9) |
|  |  | n=497 | n=276 | n=178 | n=16 | n=27 |
| Head circumference, cm |  | 34.2 (1.2) | 34.4 (0.9) | 34.0 (1.3) | 35.2 (3.3) | 34.0 (1.3) |
|  |  | n=494 | n=272 | n=178 | n=19 | n=25 |
| Cord blood serum zinc level, µg/dL***^b^*** |  | 79.2 (18.3) | 80.9 (17.5) | 80.2 (18.5) | 71.2 (19.6) | 60.7 (11.3) |

*^a^* All values are Mean (SD) except where specified

*^b^* To convert zinc in µg/dL to SI unit (µmol/L) multiply by 0.153

SGA, small for gestational age (birth weight below the 10th centile or 2SD below mean for GA of reference/normal birth curves); AGA, appropriate for gestational age (birth weight between the 10^th^ and 90^th^ centile for GA of reference/normal birth curves)

**Table E. Comparison of absolute concentrations of immune markers in term AGA newborns between the hospital sites**

| Immune markers *^a^* | Total AGA group | MAMC | SJH | AIIMS | GHG |
| --- | --- | --- | --- | --- | --- |
|  |  | AGA group | AGA group | AGA group | AGA group |
| Total leukocyte count | n=452 | n=247 | n=158 | n=20 | n=27 |
| Geometric Mean (95% CI) | 16781 (16370, 17202) | 16740 (16206, 17292) | 17069 (16370, 17797) | 13367 (12152, 14704) | 18386 (16832, 20907) |
| Median (IQR) | 16650 (13725, 20125) | 16650 (13750, 20150) | 17200 (13900, 19900) | 13725 (11425, 15125) | 18950 (14700, 24100) |
| Innate immune markers |  |  |  |  |  |
| Neutrophils | n=450 | n=246 | n=157 | n=20 | n=27 |
| Geometric Mean (95% CI) | 9649 (9357, 9951) | 9912 (9535, 10302) | 9444 (8946, 9970) | 7055 (6102, 8156) | 10801 (9363, 12461) |
| Median (IQR) | 9810 (7751, 11946) | 10052 (7982, 12128) | 9730 (7700, 11946) | 7606 (5663, 8901) | 11112 (8195, 13292) |
| Dendritic cells (DCs) | n=419 | n=229 | n=144 | n=19 | n=27 |
| Geometric Mean (95% CI) | 56 (53, 59) | 52 (49, 57) | 60 (55, 66) | 57 (45, 71) | 60 (46, 79) |
| Median (IQR) | 56 (53, 82) | 54 (37, 79) | 60 (38, 86) | 60 (41, 83) | 51 (39, 92) |
| Myeloid DCs (mDCs) | n=259 | n=138 | n=93 | n=1 | n=27 |
| Geometric Mean (95% CI) | 9 (8, 9) | 8 (7, 9) | 10 (8, 11) | 11 (-, -) | 9 (7, 12) |
| Median (IQR) | 9 (6, 12) | 8 (5, 11) | 10 (7, 14) | 11 (11, 11) | 11 (6, 13) |
| Plasmacytoid DCs (pDCs) | n=259 | n=138 | n=93 | n=1 | n=27 |
| Geometric Mean (95% CI) | 9 (8, 10) | 9 (8, 10) | 9 (7, 11) | 6 (-, -) | 10 (8, 13) |
| Median (IQR) | 10 (6, 14) | 10 (6, 14) | 11 (5, 16) | 6 (6, 6) | 9 (7, 14) |
| mDC:pDC ratio | n=259 | n=138 | n=93 | n=1 | n=27 |
| Geometric Mean (95% CI) | 0.95 (0.88, 1.03) | 0.87 (0.79, 0.97) | 1.07 (0.93, 1.23) | 1.8 (-, -) | 0.94 (0.73, 1.38) |
| Median (IQR) | 0.96 (0.61, 1.42) | 0.90 (0.60, 1.37) | 1.02 (0.69, 1.60) | 1.8 (1.8, 1.8) | 0.96 (0.61, 1.48) |
| Natural Killer (NK) cells | n=451 | n=247 | n=157 | n=20 | n=27 |
| Geometric Mean (95% CI) | 768 (730, 807) | 722 (677, 771) | 855 (781, 935) | 866 (695, 1079) | 658 (519, 833) |
| Median (IQR) | 785 (522, 1124) | 709 (505, 1047) | 907 (586, 1226) | 791 (650, 1267) | 607 (412, 1150) |
| CD56bright NK cells | n=450 | n=246 | n=158 | n=19 | n=27 |
| Geometric Mean (95% CI) | 62 (59, 66) | 60 (55, 64) | 67 (61, 75) | 84 (61, 117) | 48 (38, 59) |
| Median (IQR) | 63 (42, 94) | 59 (42, 91) | 66 (44, 108) | 75 (66, 119) | 44 (34, 78) |
| Monocytes | n=447 | n=245 | n=155 | n=20 | n=27 |
| Geometric Mean (95% CI) | 1313 (1261, 1366) | 1277 (1212, 1345) | 1430 (1341, 1524) | 925 (694, 1233) | 1337 (1139, 1569) |
| Median (IQR) | 1314 (1031, 1792) | 1306 (1031, 1697) | 1466 (1063, 1921) | 966 (841, 1344) | 1413 (1058, 1662) |
| Patrolling monocytes | n=445 | n=244 | n=156 | n=18 | n=27 |
| Geometric Mean (95% CI) | 45 (43, 48) | 42 (39, 46) | 51 (46, 56) | 44 (32, 61) | 43 (35, 53) |
| Median (IQR) | 44 (31, 67) | 42 (27, 61) | 49 (34, 74) | 48 (20, 71) | 50 (33, 67) |
| Classical monocytes | n=445 | n=244 | n=156 | n=18 | n=27 |
| Geometric Mean (95% CI) | 1065 (1022, 1110) | 1032 (975, 1092) | 1142 (1067, 1222) | 881 (709, 1095) | 1080 (895, 1303) |
| Median (IQR) | 1103 (803, 1449) | 1090 (774, 1394) | 1161 (868, 1567) | 879 (775, 1201) | 1043 (880, 1435) |
| Inflammatory monocytes | n=445 | n=244 | n=156 | n=18 | n=27 |
| Geometric Mean (95% CI) | 55 (51, 58) | 52 (48, 56) | 61 (55, 68) | 42 (30, 60) | 56 (43, 74) |
| Median (IQR) | 55 (36, 84) | 52 (35, 84) | 58 (39, 95) | 39 (29, 74) | 60 (34, 81) |
| Innate like adaptive immune markers | |  |  |  |  |
| NKT cells | n=451 | n=247 | n=157 | n=20 | n=27 |
| Geometric Mean (95% CI) | 25 (23, 27) | 24 (21, 26) | 28 (24, 31) | 50 (38, 68) | 17 (14, 22) |
| Median (IQR) | 26 (14, 48) | 26 (15, 44) | 28 (14, 55) | 48 (31, 65) | 16 (10, 26) |
| iNKT cells | n=170 | n=76 | n=62 | n=5 | n=27 |
| Geometric Mean (95% CI) | 4.1 (3.6, 4.7) | 3.9 (3.2, 4.8) | 4.1 (3.2, 5.2) | 1.9 (1.0, 3.6) | 5 (4, 7) |
| Median (IQR) | 4.7 (2.5, 7.8) | 4.3 (2.2, 8.1) | 4.9 (2.4, 7.9) | 1.7 (1.6, 1.8) | 5 (4, 8) |
| TCR γδ cells | n=448 | n=245 | n=156 | n=20 | n=27 |
| Geometric Mean (95% CI) | 103 (97, 109) | 97 (90, 104) | 111 (100, 123) | 78 (60, 102) | 152 (123, 188) |
| Median (IQR) | 106 (69, 162) | 100 (66, 147) | 114 (76, 168) | 84 (49 107) | 143 (113, 212) |
| B1B cells | n=449 | n=246 | n=157 | n=19 | n=27 |
| Geometric Mean (95% CI) | 5 (5, 5) | 5 (4, 5) | 5 (5, 6) | 4 (3, 6) | 6 (4, 10) |
| Median (IQR) | 5 (3, 9) | 5 (3, 9) | 6 (3, 9) | 5 (3, 6) | 8 (3, 13) |
| Adaptive immune markers |  |  |  |  |  |
| T lymphocytes | n=451 | n=247 | n=157 | n=20 | n=27 |
| Geometric Mean (95% CI) | 2488 (2384, 2596) | 2357 (2227, 2456) | 2674 (2479, 2884) | 2415 (2142, 2722) | 2737 (2278, 3290) |
| Median (IQR) | 2598 (1863, 3394) | 2347 (1689, 2347) | 2763 (2063, 3510) | 2424 (2029, 2915) | 3046 (1882, 3880) |
| CD4+ T cells | n=450 | n=247 | n=156 | n=20 | n=27 |
| Geometric Mean (95% CI) | 1685 (1614, 1759) | 1691 (1406, 2033) | 1805 (1673, 1946) | 1746 (1541, 1978) | 1754 (1462, 2104) |
| Median (IQR) | 1784 (1277, 2317) | 1709 (1168, 2250) | 1888 (1407, 2472) | 1770 (1426, 2213) | 1944 (1283, 2445) |
| Naive CD4+ T cells | n=448 | n=246 | n=155 | n=20 | n=27 |
| Geometric Mean (95% CI) | 1628 (1559, 1700) | 1546 (1456, 1641) | 1754 (1629, 1888) | 1682 (1486, 1904) | 1663 (1363, 2030) |
| Median (IQR) | 1732 (1229, 2224) | 1620 (1145, 2163) | 1852 (1359, 2421) | 1673 (1372, 2124) | 1759 (1252, 2224) |
| T regulatory cells | n=449 | n=247 | n=155 | n=20 | n=27 |
| Geometric Mean (95% CI) | 191 (183, 199) | 186 (175, 198) | 199 (186, 213) | 197 (159, 245) | 185 (151, 225) |
| Median (IQR) | 193 (145, 259) | 188 (141, 257) | 204 (149, 265) | 195 (140, 228) | 204 (135, 250) |
| CD8+ T cells | n=447 | n=245 | n=155 | n=20 | n=27 |
| Geometric Mean (95% CI) | 835 (798, 873) | 795 (748, 844) | 896 (831, 967) | 753 (626, 906) | 941 (751, 1180) |
| Median (IQR) | 844 (610, 1176) | 800 (592, 1149) | 870 (658, 1245) | 791 (568, 935) | 948 (644, 1497) |
| Naïve CD8+ T cells | n=447 | n=245 | n=155 | n=20 | n=27 |
| Geometric Mean (95% CI) | 764.3 (730.3, 799.8) | 729 (686, 775) | 820 (760, 884) | 676 (560, 816) | 859 (681, 1083) |
| Median (IQR) | 769.2 (560.9, 1089.1) | 729 (548, 1077) | 809 (623, 1128) | 724 (520, 858) | 890 (615, 1301) |
| CD4:CD8 T cell ratio | n=447 | n=245 | n=155 | n=20 | n=27 |
| Geometric Mean (95% CI) | 2.02 (1.96, 2.09) | 2.0 (1.9, 2.1) | 2.0 (1.9, 2.2) | 2.3 (2.0, 2.7) | 1.9 (1.6, 2.5) |
| Median (IQR) | 2.02 (1.60, 2.53) | 2.0 (1.6, 2.5) | 2.0 (1.5, 2.6) | 2.3 (1.9, 2.7) | 1.7 (1.5, 2.3) |
| Naïve CD4:CD8 T cell ratio | n=447 | n=245 | n=155 | n=20 | n=27 |
| Geometric Mean (95% CI) | 2.13 (2.05, 2.21) | 2.0 (1.9, 2.1) | 2.0 (1.9, 2.1) | 2.3 (2.0, 2.6) | 1.9 (1.6, 2.2) |
| Median (IQR) | 2.14 (1.65, 2.78) | 1.9 (1.5, 2.5) | 2.0 (1.5, 2.5) | 2.2 (1.9, 2.7) | 1.8 (1.5, 2.2) |
| Naïve B cells | n=449 | n=246 | n=157 | n=19 | n=449 |
| Geometric Mean (95% CI) | 473 (444, 503) | 450 (415, 487) | 511 (458, 571) | 427 (297, 613) | 547 (428, 698) |
| Median (IQR) | 475 (314, 730) | 452 (309, 661) | 587 (330, 793) | 465 (287, 745) | 524 (319, 889) |
| CD10+ Naïve B cells | n=440 | n=240 | n=155 | n=19 | n=440 |
| Geometric Mean (95% CI) | 116 (108, 124) | 109 (99, 119) | 124 (109, 141) | 119 (83, 171) | 122 (95, 157) |
| Median (IQR) | 123 (73, 186) | 115 (73, 174) | 133 (73, 210) | 132 (69, 220) | 102 (83, 181) |
| Immunoglobulin |  |  |  |  |  |
| IgM, mg/dL*^b^* | n=468 | n=256 | n=166 | n=17 | n=29 |
| Geometric Mean (95% CI) | 7.05 (6.69, 7.44) | 7.3 (6.8, 7.8) | 6.7 (6.1, 7.3) | 6.7 (4.7, 9.6) | 7 (6, 9) |
| Median (IQR) | 7.15 (4.52, 10.56) | 7.3 (4.5, 10.9) | 6.7 (4.5, 9.7) | 4.9 (4.5, 9.7) | 8 (5, 12) |
| IgA, mg/dL *^b^* | n=459 | n=251 | n=160 | n=19 | n=29 |
| Geometric Mean (95% CI) | 0.71 (0.66, 0.75) | 0.55 (0.51, 0.59) | 1.03 (0.93, 1.14) | 0.80 (0.55, 1.17) | 0.78 (0.65, 0.95) |
| Median (IQR) | 0.69 (0.44, 1.11) | 0.56 (0.36, 0.84) | 1.04 (0.67, 1.46) | 0.69 (0.42, 1.48) | 0.72 (0.56, 1.08) |

*^a^* All immune marker (IM) variables have been log transformed

*^b,^* To convert IgM and IgA in mg/dL to SI unit (mg/L) multiply by 10

SGA, small for gestational age (birth weight below the 10^th^ centile or 2SD below mean for GA of reference/normal birth curves); AGA, appropriate for gestational age (birth weight between the 10^th^ and 90^th^ centile for GA of reference/normal birth curves); ERC, exponentiated regression coefficient, ordinary least square (OLS) regression analysis after log transformation of dependent variable (immune marker); 95% CI, 95 percent confidence interval.

**Table F. Comparison of relative frequencies of immune markers in term AGA newborns between the hospital sites**

| Immune markers *^a^* | AGA group | MAMC | SJH | AIIMS | GHG |
| --- | --- | --- | --- | --- | --- |
|  |  | AGA group | AGA group | AGA group | AGA group |
| Innate immune markers |  |  |  |  |  |
| Neutrophils | n=450 | n=246 | n=157 | n=20 | n=27 |
| Geometric Mean (95% CI) | 57.5 (56.6, 58.4) | 59 (58, 60) | 55.3 (53.7, 56.9) | 57.5 (56.6, 58.4) | 58.7 (55.8, 61.9) |
| Median (IQR) | 58.4 (52.6, 64.7) | 60 (55, 66) | 56.3 (50.2, 63.1) | 54.5 (47.6, 57.7) | 58.4 (53.3, 65.9) |
| Dendritic cells (DCs) | n=419 | n=229 | n=144 | n=19 | n=27 |
| Geometric Mean (95% CI) | 0.33 (0.32, 0.35) | 0.32 (0.30, 0.34) | 0.35 (0.32, 0.37) | 0.4 (0.3, 0.5) | 0.33 (0.26, 0.41) |
| Median (IQR) | 0.34 (0.24, 0.46) | 0.32 (0.23, 0.45) | 0.35 (0.27, 0.46) | 0.4 (0.3, 0.7) | 0.32 (0.23, 0.43) |
| Myeloid DCs (mDCs) | n=259 | n=138 | n=93 | n=1 | n=27 |
| Geometric Mean (95% CI) | 14.2 (13.2, 15.2) | 14 (13, 15) | 14.3 (12.7, 16.2) | 12.8 (-, -) | 16 (12, 20) |
| Median (IQR) | 14.5 (9.7, 22.0) | 14 (9, 22) | 15.0 (10.0, 22.0) | 12.8 (12.8, 12.8) | 17 (10, 24) |
| Plasmacytoid DCs (pDCs) | n=259 | n=138 | n=93 | n=1 | n=27 |
| Geometric Mean (95% CI) | 14.9 (13.8, 16.2) | 16 (14, 17) | 13.4 (11.4, 15.7) | 7.3 (-, -) | 17 (13, 21) |
| Median (IQR) | 15.8 (10.2, 24.4) | 15 (11, 25) | 15.6 (8.8, 23.8) | 7.3 (7.3, 7.3) | 18 (10, 28) |
| Natural Killer (NK cells) | n=451 | n=247 | n=157 | n=20 | n=27 |
| Geometric Mean (95% CI) | 4.57 (4.36, 4.79) | 4.3 (4.1, 4.6) | 5.0 (4.6, 5.4) | 6.4 (5.2, 7.9) | 3.6 (2.9, 4.4) |
| Median (IQR) | 4.66 (3.36, 6.47) | 4.3 (3.1, 6.2) | 5.4 (3.7, 7.4) | 6.5 (4.3, 8.5) | 3.4 (2.4, 5.3) |
| CD56bright NK cells | n=450 | n=246 | n=158 | n=19 | n=27 |
| Geometric Mean (95% CI) | 8.09 (7.73, 8.47) | 8.2 (7.8, 8.7) | 7.9 (7.3, 8.6) | 8.9 (6.8, 11.7) | 7.2 (6.1, 8.6) |
| Median (IQR) | 8.00 (6.10, 11.0) | 8.2 (6.4, 10.8) | 7.6 (5.8, 11.5) | 9.2 (6.8, 11.3) | 7.3 (5.7, 10.0) |
| Monocytes | n=447 | n=245 | n=155 | n=20 | n=27 |
| Geometric Mean (95% CI) | 7.82 (7.59, 8.05) | 7.6 (7.3, 7.9) | 8.4 (8.0, 8.8) | 6.9 (5.3, 9.1) | 7.3 (6.7, 7.9) |
| Median (IQR) | 8.0 (6.52, 9.54) | 7.8 (6.4, 9.1) | 8.7 (6.8, 10.2) | 8.1 (6.4, 9.7) | 7.1 (6.2, 8.6) |
| Patrolling monocytes | n=445 | n=244 | n=156 | n=18 | n=27 |
| Geometric Mean (95% CI) | 3.71 (3.52, 3.90) | 3.6 (3.3, 3.9) | 3.9 (3.6, 4.2) | 4.3 (3.4, 5.4) | 3.5 (2.8, 4.4) |
| Median (IQR) | 3.76 (2.76, 5.08) | 3.6 (2.6, 5.0) | 4.0 (2.9, 5.3) | 4.4 (3.5, 5.0) | 4.2 (2.4, 5.2) |
| Classical monocytes | n=445 | n=244 | n=156 | n=18 | n=27 |
| Geometric Mean (95% CI) | 87.6 (87.0, 88.1) | 88 (87, 89) | 87.3 (86.4, 88.2) | 86.0 (83.2, 88.9) | 88 (86, 90) |
| Median (IQR) | 88.7 (85.1, 91.5) | 89 (85, 92) | 88.2 (85.0, 91.1) | 88.2 (82.9, 90.2) | 88 (86, 91) |
| Inflammatory monocytes | n=445 | n=244 | n=156 | n=18 | n=27 |
| Geometric Mean (95% CI) | 4.49 (4.26, 4.74) | 4.4 (4.1, 4.7) | 4.7 (3.6, 6.2) | 4.1 (3.1, 5.5) | 4.6 (3.8, 5.5) |
| Median (IQR) | 4.6 (3.07, 6.66) | 4.4 (3.0, 6.7) | 4.7 (3.1, 7.3) | 4.3 (3.0, 5.6) | 4.4 (3.4, 6.3) |
| Innate like adaptive immune markers | |  |  |  |  |
| NKT cells | n=451 | n=247 | n=157 | n=20 | n=27 |
| Geometric Mean (95% CI) | 1.01 (0.93, 1.10) | 1.0 (0.9, 1.1) | 1.0 (0.9, 1.2) | 2.1 (1.5, 2.8) | 0.6 (0.5, 0.8) |
| Median (IQR) | 1.05 (0.56, 1.86) | 1.1 (0.6, 1.8) | 1.0 (0.6, 1.9) | 2.0 (1.3, 2.8) | 0.6 (0.5, 0.9) |
| iNKT cells | n=170 | n=76 | n=62 | n=5 | n=27 |
| Geometric Mean (95% CI) | 0.28 (0.25, 0.32) | 0.36 (0.29, 0.44) | 0.24 (0.20, 0.28) | 0.36 (0.16, 0.82) | 0.21 (0.18, 0.26) |
| Median (IQR) | 0.26 (0.17, 0.43) | 0.33 (0.21, 0.58) | 0.23 (0.15, 0.32) | 0.28 (0.28, 0.53) | 0.22 (0.17, 0.28) |
| TCR γδ cells | n=448 | n=245 | n=156 | n=20 | n=27 |
| Geometric Mean (95% CI) | 4.14 (3.96, 4.34) | 4.1 (3.9, 4.4) | 4.1 (3.8, 4.5) | 3.3 (2.6, 4.2) | 5.5 (4.8, 6.5) |
| Median (IQR) | 4.24 (3.08, 5.52) | 4.2 (3.0, 5.7) | 4.2 (3.0, 5.3) | 3.8 (2.3, 4.3) | 5.2 (4.2, 7.4) |
| B1B cells | n=449 | n=246 | n=157 | n=19 | n=27 |
| Geometric Mean (95% CI) | 1.02 (0.97, 1.08) | 1.04 (0.96, 1.12) | 0.96 (0.87, 1.06) | 0.96 (0.69, 1.34) | 1.4 (1.1, 1.7) |
| Median (IQR) | 1.08 (0.77, 1.5) | 1.10 (0.77, 1.50) | 1.00 (0.75, 1.40) | 0.95 (0.55, 1.40) | 1.5 (1.2, 1.9) |
| Adaptive immune markers |  |  |  |  |  |
| T lymphocytes | n=451 | n=247 | n=157 | n=20 | n=27 |
| Geometric Mean (95% CI) | 14.8 (14.2, 15.4) | 14.1 (13.4, 14.8) | 15.7 (14.6, 16.8) | 17.9 (15.4, 20.9) | 14.9 (12.7, 17.6) |
| Median (IQR) | 15.4 (11.5, 20.0) | 14.6 (10.9, 19.0) | 16.2 (12.4, 21.0) | 18.3 (13.4, 23.9) | 15.4 (11.2, 20.2) |
| CD4+ T cells | n=450 | n=247 | n=156 | n=20 | n=27 |
| Geometric Mean (95% CI) | 10.0 (9.6, 10.5) | 9.6 (9.1, 10.1) | 10.6 (9.8, 11.4) | 12.1 (11.3, 15.1) | 9.5 (8.0, 11.4) |
| Median (IQR) | 10.4 (7.7, 13.7) | 10.1 (7.3, 12.9) | 11.0 (8.4, 14.4) | 14.7 (9.5, 16.1) | 9.8 (6.8, 12.3) |
| Naive CD4+ T cells | n=448 | n=246 | n=155 | n=20 | n=27 |
| Geometric Mean (95% CI) | 96.5 (96.4, 96.7) | 96.6 (96.3, 96.8) | 97 (96, 97) | 96 (96, 97) | 96.6 (96.0, 97.2) |
| Median (IQR) | 97.0 (96.0, 98.0) | 97.0 (96.0, 98.0) | 97.0 (95.0, 98.0) | 96.0 (95.0, 97.0) | 96.4 (95.0, 98.0) |
| T regulatory cells | n=449 | n=247 | n=155 | n=20 | n=27 |
| Geometric Mean (95% CI) | 1.14 (1.09, 1.19) | 1.1 (1.0, 1.2) | 1.17 (1.09, 1.25) | 1.48 (1.18 , 1.86) | 1.0 (0.9, 1.2) |
| Median (IQR) | 1.14 (0.89, 1.55) | 1.1 (0.9, 1.5) | 1.18 (0.89, 1.60) | 1.33 (1.06 , 1.74) | 1.0 (0.8, 1.3) |
| CD8+ T cells | n=447 | n=245 | n=155 | n=20 | n=27 |
| Geometric Mean (95% CI) | 4.98 (4.78, 5.19) | 4.8 (4.5, 5.0) | 5.3 (4.9, 5.6) | 5.6 (4.7 , 6.8) | 5.1 (4.3, 6.1) |
| Median (IQR) | 5.0 (3.78, 6.7) | 4.8 (3.5, 6.5) | 5.2 (4.1, 6.7) | 5.7 (4.2 , 7.4) | 5.9 (3.7, 7.2) |
| Naïve CD8+ T cells | n=447 | n=245 | n=155 | n=20 | n=27 |
| Geometric Mean (95% CI) | 91.1 (90.8, 93.4) | 91.7 (91.1, 92.4) | 91 (91, 92) | 90 (86, 93) | 91.2 (89.1, 93.4) |
| Median (IQR) | 93 (89.3, 95.1) | 93.0 (90.0, 95.1) | 93 (89, 95) | 92 (89, 95) | 93.2 (89.0, 95.0) |
| B lymphocytes | n=449 | n=246 | n=157 | n=19 | n=27 |
| Geometric Mean (95% CI) | 2.7 (2.6, 2.9) | 2.6 (2.4, 2.8) | 2.8 (2.6, 3.1) | 3.3 (2.4, 4.6) | 3.1 (2.5, 3.9) |
| Median (IQR) | 2.8 (2.0, 4.1) | 2.7 (2.0, 3.9) | 2.9 (2.0, 4.3) | 3.5 (2.4, 5.9) | 3.1 (2.0, 5.1) |
| Naïve B cells | n=449 | n=246 | n=157 | n=19 | n=27 |
| Geometric Mean (95% CI) | 96.6 (96.4, 96.8) | 96.6 (96.3, 96.9) | 97 (97, 97) | 97 (96, 97) | 95.0 (94.0, 96.0) |
| Median (IQR) | 97.0 (95.7, 98.0) | 97.0 (96.0, 98.0) | 97 (96, 98) | 97 (95, 98) | 95.7 (93.5, 96.8) |
| CD10+ Naïve B cells | n=440 | n=240 | n=155 | n=19 | n=26 |
| Geometric Mean (95% CI) | 23.6 (22.8, 24.4) | 23.6 (22.5, 24.7) | 24 (22, 25) | 27 (23, 32) | 21.2 (18.6, 24.2) |
| Median (IQR) | 23.5 (19.1, 30.5) | 24.0 (18.9, 30.8) | 23 (19, 31) | 26 (20, 34) | 22.6 (17.7, 27.1) |

*^a^* All immune marker variables have been log transformed

SGA, small for gestational age (birth weight below the 10^th^ centile or 2SD below mean for GA of reference/normal birth curves); AGA, appropriate for gestational age (birth weight between the 10^th^ and 90^th^ centile for GA of reference/normal birth curves); ERC, exponentiated regression coefficient, ordinary least square (OLS) regression analysis after log transformation of dependent variable (immune marker); 95% CI, 95 percent confidence interval.
